# Anti-protozoal activity and metabolomic analyses of *Cichorium intybus* L. against *Trypanosoma cruzi*

**DOI:** 10.1016/j.ijpddr.2022.08.002

**Published:** 2022-08-13

**Authors:** Miguel Peña-Espinoza, Yeambell Romero-Uzqueda, Angela H. Valente, Matthew de Roode, Henrik T. Simonsen, Stig M. Thamsborg, Andrew R. Williams, Rodrigo López-Muñoz

**Affiliations:** a Instituto de Farmacologia y Morfofisiologia, Facultad de Ciencias Veterinarias, Universidad Austral de Chile, Valdivia, Chile; b Institute of Parasitology, Department of Pathobiology, University of Veterinary Medicine Vienna, 1210 Vienna, Austria; c Department of Veterinary and Animal Sciences, Faculty of Health and Medical Sciences, University of Copenhagen, 1870 Frederiksberg C, Denmark; d Sensus B.V., 4704 RA Roosendaal, the Netherlands; e Department of Biotechnology and Biomedicine, Technical University of Denmark, 2800 Kongens Lyngby, Denmark

**Keywords:** Chagas disease, *Trypanosoma cruzi*, Anti-protozoal, *Cichorium intybus*, Sesquiterpene lactones, Metabolomics

## Abstract

Chagas disease, caused by the protozoa *Trypanosoma cruzi*, is a potentially life-threatening parasitic zoonosis infecting 6–7 million people worldwide, mainly in Latin America. Due to the limited numbers of drugs available against this neglected disease and their frequent adverse effects, novel anti-chagasic agents are urgently needed. *Cichorium intybus* L. (chicory) is a bioactive plant with potent activity against parasitic nematodes, but its effects on protozoans are poorly known and no studies have explored its trypanocidal potential. Here, we investigated the activity of *C. intybus* against extracellular and intracellular stages of *T. cruzi*, including the prediction of trypanocidal compounds by metabolomic analyses and bioactivity-based molecular networking. Purified *C. intybus* extracts were prepared from leaves and roots of five *C. intybus* cultivars (cv. ‘Benulite’, ‘Goldine’, ‘Larigot’, ‘Maestoso’ and ‘Spadona’). All *C. intybus* extracts induced concentration-dependent effects against *T. cruzi* trypomastigotes. *C. intybus* leaf extracts had higher trypanocidal selectivity and lower cytotoxicity on mammalian cells than root extracts. The leaf extract of *C. intybus* cv. Goldine also significantly reduced the number of mammalian cells infected with *T. cruzi* amastigotes. Metabolomic and bioactivity-based molecular networking analyses revealed 11 compounds in *C. intybus* leaves strongly linked with activity against trypomastigotes, including the sesquiterpene lactone lactucin, and flavonoid- and fatty acid-derivatives. Furthermore, seven distinct *C. intybus* molecules (including two sesquiterpene lactone-derivatives) were predicted to be involved in reducing the number of mammalian cells infected with amastigotes. This is the first report of the anti-protozoal activity of *C. intybus* against trypanosomatid parasites and expands our understanding of the anti-parasitic effects of this plant and its bioactive metabolites. Further studies to elucidate the anti-protozoal compound(s) in *C. intybus* and their mode(s) of action will improve our knowledge of using this bioactive plant as a promising source of novel broad-spectrum anti-parasitic compounds with associated health benefits and biomedical potential.

## Graphical abstract

**Figure:**
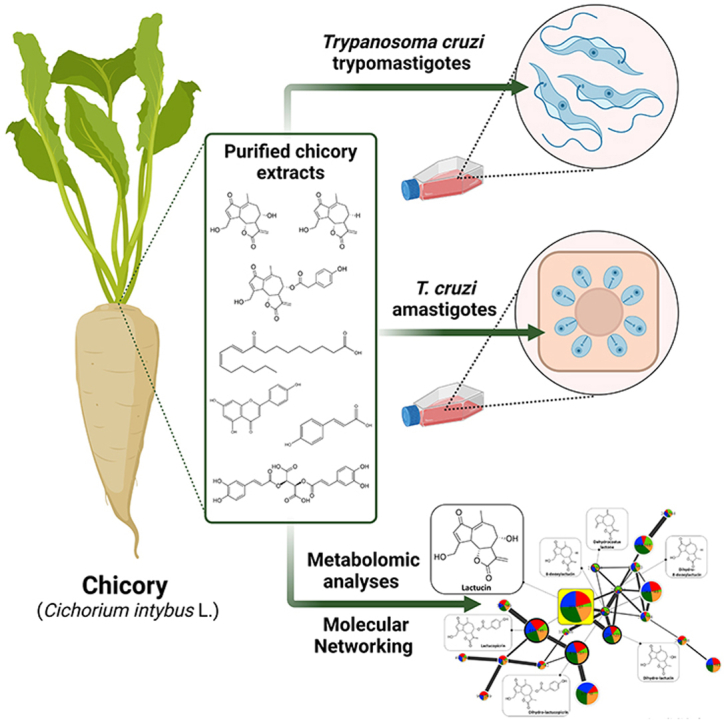

## 1 Introduction

Chagas disease is a vector-borne zoonosis and potentially life-threatening disease caused by the protozoan parasite *Trypanosoma cruzi*. Approximately 6–7 million people are infected with *T. cruzi* worldwide, mainly in Latin America, and with an increasing number of cases also been reported in the US, Canada, Europe, Australia and Japan (Schmunis and Yadon, 2010; Pérez-Molina and Molina, 2018; Elkheir et al., 2021). Chagas disease is a neglected tropical disease, and only two effective anti-chagasic drugs are available; benznidazole and nifurtimox (World Health Organization, 2017). However, these drugs have limited activity against chronic infections and induce frequent adverse effects in up to 50% of treated patients, leading to discontinued treatment (World Health Organization, 2017; Pérez-Molina and Molina, 2018). Therefore, the development of novel agents against *T. cruzi* is urgently needed. In the search for new anti-chagasic drugs, natural products have been increasingly explored as potential sources of novel trypanocidal molecules (Bermudez et al., 2016; Cockram and Smith, 2018; Ribeiro et al., 2020). *Cichorium intybus* L. (Asteraceae, common name “chicory”) is a perennial plant distributed worldwide and cultivated as a multi-purpose crop and as a medicinal plant in some countries (Street et al., 2013; Bogdanović et al., 2019). *Cichorium intybus* is known to synthesize several natural bioactive metabolites with associated health benefits, such as sesquiterpene lactones (SL), polyphenols and fatty acids, which are detectable in leaves and roots (Bogdanović et al., 2019; Peña-Espinoza et al., 2020; Janda et al., 2021). A growing scientific interest has focused on the study of *C. intybus* compounds and their biological activities, including anti-inflammatory and anti-parasitic effects (Bischoff et al., 2004; Peña-Espinoza et al., 2018; Baixinho et al., 2021; Perović et al., 2021). During the past 20 years, *C. intybus* has been intensively investigated for its activity against parasitic helminths in animals, which has been linked to its content of SL (Peña-Espinoza et al., 2018). Previous *in vitro* studies have reported that SL-rich *C. intybus* extracts can induce potent anthelmintic effects against parasitic nematodes (Foster et al., 2011; Peña-Espinoza et al., 2015, 2017, 2020; Williams et al., 2016). Recently, the anthelmintic activity of pure SL from *C. intybus* against parasitic nematodes has been confirmed by molecular networking and bioactivity-guided fractionation (Valente et al., 2021). Nevertheless, little is known about the potential of *C. intybus* and its bioactive compounds against other major pathogens of medical or veterinary importance, such as protozoan parasites.

To date, only two studies have explored the anti-protozoal activity of *C. intybus*. Bischoff et al. (2004) reported direct *in vitro* effects of purified SL (lactucin and lactucopicrin) from *C. intybus* roots against the malarial parasite *Plasmodium falciparum*, with total inhibition of parasite growth at 10 and 50 μg/mL of lactucin and lactucopicrin, respectively. More recently, Woolsey et al. (2019) described the *in vitro* activity of purified *C. intybus* leaf and root extracts against the zoonotic protozoa *Cryptosporidium parvum*, demonstrating a concentration-dependent inhibition of parasite growth. However, no studies have evaluated the anti-parasitic activity of *C. intybus* against *T. cruzi* or other trypanosomatid parasites. The objectives of the present study were to investigate the anti-protozoal activity of *C. intybus* against extracellular and intracellular stages of *T. cruzi* and their cytoxicity in mammalian host cells, and to carry out metabolomic analyses and bioactivity-based molecular networking of the tested fractions to facilitate prediction and identification of trypanocidal compounds.

## 2 Materials and methods

### 2.1 Plant material and extraction

Leaf and root fresh material from five different *C. intybus* cultivars was used for preparation of purified extracts, evaluation of anti-protozoal and cytotoxicity activities and metabolomic analyses. The sources of plant material, sample collection and preparation of purified extracts are described in details in Valente et al. (2021). Briefly, fresh leaves and roots were collected from four industrial *C. intybus* cultivars grown by Sensus B.V. (Roosendaal, The Netherlands): cultivar (cv.) ‘Benulite’, cv. ‘Goldine’, cv. ‘Larigot’ and cv. ‘Maestoso’ (sampled in September 2016 from the test field of Sensus, Colijnsplaat, The Netherlands). Fresh leaves and roots were also sampled from *C. intybus* cv. ‘Spadona’, grown as a pure sward at the experimental farm of the University of Copenhagen (Taastrup, Denmark), in September 2017. Collected plant material (leaves and roots) from the five chicory cultivars was subjected to an extraction method optimized for recovery of SL described by Valente et al. (2021). In brief, freeze-dried plant material was dissolved in methanol/H_2_O (4/1; v/v) with 2% (v/v) formic acid and extracted for 10 min in sonication at room temperature. Resulting crude extracts were recovered and dried under reduced pressure followed by freeze-drying. Dried crude extracts were incubated overnight with Viscozyme® L cellulolytic enzyme mixture (Sigma-Aldrich) for removal of bound sugars, and *C. intybus* metabolites were recovered with ethyl acetate. Resulting ethyl acetate extracts were dried under reduced pressure, dissolved in 14% methanol in dichloromethane and purified by solid-phase extraction (SPE). After SPE, collected liquid fractions were dried under nitrogen flux and the resulting purified extracts were weighed and stored at −20 °C until *in vitro* studies and metabolomic analyses. A total of 10 purified extracts (from root and leaf material of the five *C. intybus* cultivars described above) were selected and used in the present study. Separate aliquots from the same extracts were dissolved in either 100% dimethyl sulfoxide (DMSO) at a stock concentration of 100 mg dry extract/mL (for *in vitro* assays) or 100% methanol at a concentration of 10 mg dry extract/mL (for metabolite profiling).

### 2.2 Metabolite profiling of *C. intybus* extracts by untargeted metabolomics

The metabolite profile of all ten purified *C. intybus* extracts was analysed by an untargeted metabolomic platform as described in Valente et al. (2021). Briefly, purified *C. intybus* extracts (10 mg dry extract/mL in methanol) were analysed in an ultra-high performance liquid chromatography-high resolution mass spectrometry (UHPLC-HRMS) platform (Agilent Infinity 1290 UHPLC system QTOF, coupled with an Agilent 6545 QTOF MS; Agilent Technologies, Santa Clara, CA, USA). Resulting LC-MS/MS chromatograms from every purified *C. intybus* extract were converted to mzXML files and pre-processed using MZmine. For each extract, a table containing all extracted MS features (.csv file) associated with their MS fragmentation spectra (.mgf file) was obtained, and all data was submitted for compound identification and bioactivity-based molecular networking analyses in the Global Natural Products Social (GNPS)'s spectral libraries and Molecular Networking Platform (https://gnps.ucsd.edu/; Wang et al., 2016).

### 2.3 Parasite and mammalian cell cultures

*Trypanosoma cruzi* (Y strain, Discrete Type Unit II) cultures were maintained in infected Vero cells (*Chlorocebus sabaeus*; ATCC® CCL-81) following the method described by Cortes et al. (2015), with minor modifications. Vero cells were cultivated in culture flasks with RPMI 1640 supplemented with 2% (v/v) foetal bovine serum (FBS), penicillin 100 U/mL and streptomycin 100 μg/mL (37 °C, 5% CO_2_). Vero cells were then infected with *T. cruzi* trypomastigotes at 4:1 density (trypomastigote:cell), and 4–5 days post-infection, infected cells released *T. cruzi* trypomastigotes to the culture media that were collected and immediately used for *in vitro* assays (described in section 2.4) or to infect new Vero cells to maintain *T. cruzi* cultures. Uninfected Vero cells were cultivated separately for cytotoxicity studies (section 2.5).

### 2.4 Anti-protozoal activity of *C. intybus* against extracellular *T. cruzi* trypomastigotes

First, we assessed the direct anti-protozoal activity of all ten purified *C. intybus* extracts (tested individually) on isolated *T. cruzi* trypomastigotes obtained as described in section 2.3. Trypomastigotes (10^6^ trypomastigotes/well) were placed in black 96-well plates and incubated with decreasing concentrations of each *C. intybus* extract (dissolved in DMSO) for 24 h in RPMI supplemented with antibiotics (37 °C, 5% CO_2_). Final concentrations tested in triplicates ranged from 100 to 6.3 μg C*. intybus* extract/mL (0.1% DMSO in all wells). Benznidazole (BNZ) was used as positive control and tested at a single concentration of 260 μg/mL (i.e. 100 μM BNZ) to enable the complete inhibition of trypomastigote viability, based on previous results with *T. cruzi* Y strain (Torchelsen et al., 2021). Positive controls (260 μg BNZ/mL, 0.1% DMSO in well) and negative controls (0.1% DMSO) were run in triplicates at similar conditions. After 24 h of incubation, the viability of exposed trypomastigotes was evaluated using the resazurin reduction assay (Rolón et al., 2006), which measures the reduction of resazurin (non-fluorescent) to resorufin (highly fluorescent) by metabolically active cells and is directly proportional to the number of viable trypomastigotes (O'Brien et al., 2000; Rolón et al., 2006). Briefly, 20 μL of resazurin (1 mM) were added to each well, and after 4 h of incubation (37 °C, 5% CO_2_), the reduction of resazurin into resorufin by viable parasites was measured at 560 nm (excitation) and 590 nm (emission) in an automated Varioskan Flash reader (Thermo Fisher Scientific, USA). To evaluate the potential confounding reduction of resazurin by the tested *C. intybus* extracts (and not by *T. cruzi*), blank wells containing the same tested concentrations of extracts and controls, but without trypomastigotes, were incubated under the conditions as described above and evaluated with the resazurin reduction assay. The resulting relative fluorescent units (RFU) were normalized to their negative controls and expressed as parasite viability percentages. Three independent viability experiments with *T. cruzi* trypomastigotes were performed.

### 2.5 Cytotoxicity of *C. intybus* on uninfected Vero cells

We then evaluated the potential cytotoxicity of *C. intybus* on mammalian cells by exposing uninfected Vero cells to decreasing concentrations of all ten purified *C. intybus* extracts (tested individually). Uninfected Vero cells were placed in black 96-well plates (5 × 10^4^ cells/well) and incubated in RPMI supplemented with antibiotics (37 °C, 5% CO_2_). After overnight incubation to allow the attachment of Vero cells to the plates, the uninfected cells were exposed to decreasing concentrations of all *C. intybus* extracts in triplicates (100, 50 or 0 μg extract/mL; 0.1% DMSO in well). Vero cells exposed to BNZ (260 μg/mL) and DMSO (0.1%) in triplicates were run as positive and negative controls, respectively. After 24 h incubation, the viability of exposed Vero cells was measured by the resazurin reduction assay as described in section 2.4. The resulting RFU were normalized to the negative controls and expressed as cell viability percentages. Three independent viability experiments with uninfected Vero cells were performed.

### 2.6 Anti-protozoal activity of *C. intybus* against intracellular *T. cruzi* amastigotes

We further assessed the trypanocidal activity of *C. intybus* against *T. cruzi* amastigotes, the replicative intracellular stage of the parasite. The leaf extracts from the five *C. intybus* cultivars were selected for these experiments based on their higher selectivity towards *T. cruzi* (in comparison with the root extracts). We evaluated the effects of these five leaf *C. intybus* extracts on intracellular *T. cruzi* amastigotes using an *in vitro* infection model described by Cortes et al. (2015), with modifications. Briefly, sterile round coverslides (15 mm diameter) were added to each well of 24-well plates, followed by seeding of uninfected Vero cells (10^4^ cells/well) and *T. cruzi* trypomastigotes (100:1 density, trypomastigote:cell) in RPMI supplemented with 2% FBS and antibiotics. Parasite invasion of Vero cells was allowed for 48 h (37 °C, 5% CO_2_), followed by removal of the supernatant and replacement by fresh medium containing *C. intybus* extracts (final concentration in well: 10 μg extract/mL, 0.1% DMSO). BNZ was used as positive control and tested at a single concentration of 26 μg/mL (i.e. 10 μM BNZ; 0.1% DMSO) to enable the complete inhibition of intracellular amastigotes, based on previous results with *T. cruzi* Y strain (Grecco et al., 2017). Infected cells exposed to DMSO (0.1%) were incubated as negative controls. After 48 h incubation, exposed cells (attached to the upper side of the coverslides) were washed with sterile PBS and fixed in cold methanol (70%) for 12 h at 4 °C. Then, fixed cells were incubated with DAPI (DAPI:PBS, 1:50,000; NucBlue, Molecular Probes, USA) for 20 min in the dark (room temperature) to selectively stain the DNA of Vero cells and *T. cruzi* amastigotes. The coverslide containing stained cells were transferred into microscope slides and photographed using a fluorescence microscope (Leica DMi8) at 358 nm (excitation) and 461 nm (emission). Three images were randomly obtained for each cover slide (i.e. treatment) at 20 × magnification, and the images were automatically analysed using the software Image J (1.52) to quantify the total number of Vero cells, the number of Vero cells infected with *T. cruzi* amastigotes and the number of *T. cruzi* amastigotes per infected cell.

### 2.7 Bioactivity-based molecular networking for prediction of trypanocidal compounds in *C. intybus*

The potential trypanocidal compounds in the tested purified *C. intybus* extracts were predicted by bioactivity-based molecular networking analyses (Wang et al., 2016; Nothias et al., 2018). Molecular networks represent the associations of molecules in related MS data by alignment of different compounds' mass spectrum based on their similar fragmentation patterns (Wang et al., 2016). Bioactivity-based molecular networking integrates the molecular networks of these related MS data (e.g. different extracts from the same plant species) and their bioactivities (e.g. *in vitro* anti-parasitic activity of each extract) to predict “bioactivity scores” for each compound in all analysed extracts (Nothias et al., 2018; Peña-Espinoza et al., 2020). A bioactivity score is the correlation between the relative quantification of a single compound in an extract (expressed as individual peak area) and the bioactivity of this extract (e.g. EC_50_ value), thus predicting the probability of a compound being active in a given biological system (Nothias et al., 2018). In the present study, bioactivity-based molecular networking analyses were performed as described by Valente et al. (2021). In brief, the LC-MS/MS chromatograms from the five *C. intybus* leaf extracts that were tested on *T. cruzi* trypomastigotes and amastigotes (higher selectivity index), were pre-processed with MZmine. All extracted mass spectral features and aggregated MS2 fragmentation spectra were submitted for compound identification and molecular networking at the GNPS platform using GNPS's spectral libraries and feature-based mass spectral molecular networking online workflow (https://gnps.ucsd.edu/; Nothias et al., 2020). The resulting molecular network representing the relationship between all compounds detected in the tested purified *C. intybus* extracts is accessible at:

https://gnps.ucsd.edu/ProteoSAFe/status.jsp?task=6fb25560f4b948fa9ac0406fdabdb321.

The resulting molecular network was further processed in Cytoscape 3.8.2 (Shannon et al., 2003) to map bioactivity scores of all detected compounds in the tested extracts. Bioactivity scores were computed following the methodology and the R script provided by Nothias et al. (2018), with the modifications by Peña-Espinoza et al. (2020). Two distinct bioactivity scores were calculated for each compound, based on their predicted activity against extracellular *T. cruzi* trypomastigotes or towards intracellular *T. cruzi* amastigotes. Briefly, the individual peak area intensity of detected metabolites were normalized by adding ‘1’ ( × +1) to each peak intensity value and divided by the sum of all peak intensities in the same extract + 1 ([peak intensity + 1]/[sum of all peak intensities + 1]), followed by scaling of the samples by normalizing the peak intensity to the total ion current. Next, bioactivity scores were calculated as the Pearson correlation coefficient (*r*) between the normalized individual peak areas and the bioactivity of each *C. intybus* leaf extract in the studies with *T. cruzi* trypomastigotes ([1/EC_50_] × 10000) or in the assays with *T. cruzi* amastigotes (percentage reduction of infected Vero cells). Molecules with high bioactivity scores were defined as having a statistically significantly high positive correlation (*r* > 0.85, with significance of the correlation P < 0.03; Nothias et al., 2018). Resulting bioactivity scores (CVS format) were imported into the molecular network processed in Cytoscape (see above) to construct the bioactivity-based molecular network for all compounds detected in the purified *C. intybus* extracts.

### 2.8 Statistical analyses

In the anti-protozoal studies of *C. intybus* against extracellular *T. cruzi* trypomastigotes, the effective concentrations of each tested extract able to inhibit the viability in 50% of exposed parasites (EC_50_) were calculated. Tested extract concentrations were log-transformed, and parasite viability percentages obtained for each condition (nine replicates per concentration from three independent assays) were analysed by non-linear (least squares) regression using the model *log (inhibitor) vs. response - variable slope* in GraphPad Prism®7.03 (GraphPad Software, San Diego, CA, USA). The R squared measure of goodness of fit (R^2^) was calculated for each concentration-response curve. In the cytotoxicity assays with uninfected Vero cells, the cytotoxic concentrations of each *C. intybus* extract able to reduce the viability in 50% of exposed cells (CC_50_) were calculated. Tested extract concentrations were log-transformed and cell viability percentages (nine replicates per concentration from three independent cytotoxicity studies) were analysed by non-linear (least squares) regression as described above. The selectivity index (SI) of each *C. intybus* extract was calculated as the ratio of their CC_50_ values on uninfected Vero cells to their EC_50_ values on *T. cruzi* trypomastigotes (SI: CC_50_ Vero cells/EC_50_ *T. cruzi* trypomastigotes). Statistical differences between EC_50_ and CC_50_ obtained with leaf vs. root extracts from the same *C. intybus* cultivar were analysed by extra sum-of-squares F test with a null hypothesis of equal EC/CC_50_. In the anti-protozoal studies of *C. intybus* with intracellular *T. cruzi* amastigotes, the number of infected cells and the number of amastigotes per infected cell after exposure to purified leaf extracts or BNZ were compared with negative controls (DMSO) by one-way ANOVA with Dunnett's post-test. The percentage reduction of infected Vero cells with *T. cruzi* amastigotes was also calculated for each purified *C. intybus* leaf extract. A value of P < 0.05 was considered significant.

## 3 Results

### 3.1 Metabolite profiling of purified *C. intybus* extracts by untargeted metabolomics

**Fig. 1:**
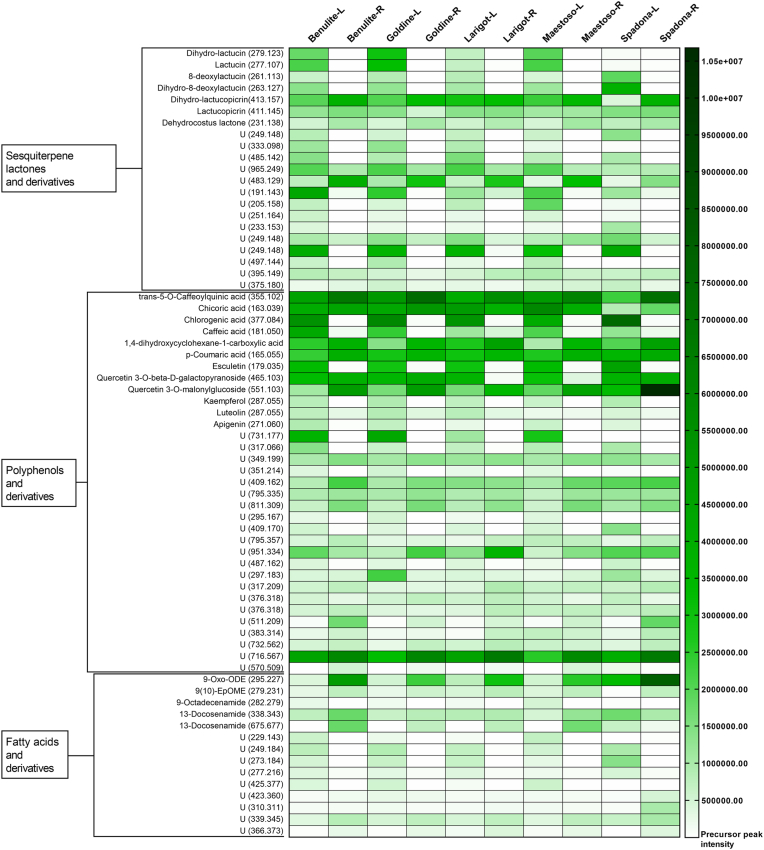
Metabolite profile and relative quantification of compounds from the three classes of compounds detected in purified *Cichorium intybus* extracts evaluated for trypanocidal activity. Leaf (L) and root (C) extracts from *C. intybus* (cv. Benulite, cv. Goldine, cv. Larigot, cv. Maestoso and cv. Spadona) were analysed by untargeted metabolomics using UHPLC-HRMS. Metabolites were identified based on the Global Natural Product Social Molecular Networking libraries. The relative quantification of the compounds is based on the peak area of the precursor ion and is illustrated in the heatmap as shades of green (with darker green representing more abundant compounds in the extracts). Identified hit compounds or unidentified derivatives (U; no hit in the libraries but grouped within a specific class of compounds by molecular networking) are presented with their molecular weights (in Da). (For interpretation of the references to colour in this figure legend, the reader is referred to the Web version of this article.)

The metabolite profiling and relative quantification of the main compounds (according to the chemical class of compounds) identified in the ten *C. intybus* extracts are presented in Fig. 1. A summary of the total relative concentration of the main chemical classes detected in the purified extracts (based on the sum of individual peak areas) is described in Table 1. The LC/MS-MS chromatograms of each purified *C. intybus* extract are presented in Supplementary Figs. 1–10. The metabolomic analysis and compound identification in GNPS libraries revealed a different profile of identified compounds (library hits) and unidentified derivatives (no-hit in the libraries but grouped within a specific class of compounds) between the extracts. Molecules belonging to three distinct chemical classes were detected, with variations between *C. intybus* cultivars and plant parts: polyphenols, SL and fatty acids, with their derivatives (Fig. 1; Table 1). Based on the sum of peak areas of all detected compounds in the ten *C. intybus* extracts, polyphenols and their derivatives (including flavonoids) were the main identified compounds in all extracts (29.4–38.7% of total peak area), followed by SL and derivatives (8–17.5%) and fatty acids and derivatives (3.0–9.8%). Total polyphenol content was largely similar between *C. intybus* leaves and roots, except for single compounds such as chlorogenic acid, caffeic acid and esculetin (6-7-dihidroxycoumarin), which were almost only present in leaf extracts. Whereas the six major guaianolide SL (and their derivatives) synthesized by *C. intybus* were present in all extracts, and total SL was in average higher in leaves than in root material (Table 1). The individual SL lactucin, dihydro-lactucin, 8-deoxylactucin and dihydro-8-deoxylactucin were mainly detected in *C. intybus* leaf extracts but scarcely in root extracts, whereas lactucopicrin and dihydro-lactucopicrin were present at similar levels in both leaves and roots (Fig. 1). Not annotated molecules represented 44.6–54.1% of the total peak area in the ten chicory extracts (Table 1). These not annotated molecules correspond to compounds detected in the extracts by UHPLC-HRMS, but that could be neither identified in metabolomic databases (no library hit) nor grouped within a specific class of compounds by molecular networking. The relative quantification of these not annotated (unidentified) molecules in each purified *C. intybus* extract is presented in Supplementary Fig. 11.

**Table 1:** Summary of the relative quantification by class of compounds of molecules detected in leaf (L) and root (R) purified extracts from five cultivars of *Cichorium intybus* (cv. Benulite, cv. Goldine, cv. Larigot, cv. Maestoso and cv. Spadona). Purified *C. intybus* extracts were analysed by untargeted metabolomics using UHPLC-HRMS and compounds were annotated based on the Global Natural Product Social Molecular Networking libraries. Peak areas of identified compounds (library hit) and not-identified derivatives (no hit in the libraries but grouped within a specific class of compounds by molecular networking) of the same class in each extract were summed and used to calculate a peak area percentage (%) of the total peak area/extract.

	Total peak area by class of compound (%)^a^			
Plant material	Sesquiterpene lactones and derivatives	Polyphenols and derivatives	Fatty acids and derivatives	Not annotated^b^
Benulite-L	17.3	30.4	3.0	49.2
Benulite-R	8.8	29.9	7.2	54.1
Goldine-L	17.5	30.7	3.2	48.6
Goldine-R	8.9	38.7	5.3	47.1
Larigot-L	15.4	30.0	3.5	51.1
Larigot-R	9.6	32.5	5.9	52.0
Maestoso-L	16.0	29.4	3.3	51.3
Maestoso-R	10.5	32.7	6.7	50.2
Spadona-L	14.1	30.8	6.1	48.9
Spadona-R	8.0	37.5	9.8	44.6

a Sum of peak areas (%) of all precursor ions identified as sesquiterpene lactones, polyphenols, fatty acids or their derivatives (no library hit but associated to a specific class of compounds) presented in details in Fig. 1.

b Not annotated molecules (no library hit) and not grouped within any class of compound by molecular networking (presented in Supplementary Fig. 11).

### 3.2 Anti-protozoal activity of purified *C. intybus* against extracellular *T. cruzi* trypomastigotes

**Fig. 2:**
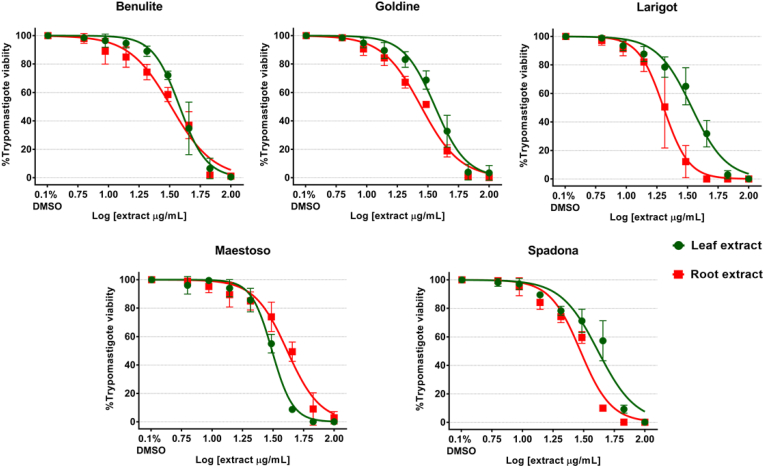
Anti-protozoal activity of purified *Cichorium intybus* extracts against extracellular *Trypanosoma cruzi* trypomastigotes. Concentration–response curves obtained in viability inhibition assays with *T. cruzi* trypomastigotes at 24 h after incubation with decreasing concentrations of purified leaf and root extracts ([log] μg extract/mL) from five *C. intybus* cultivars. Each data point in the graphs represents the mean viability percentage of 9 replicates (n = 10^6^ trypomastigotes per replicate) for each concentration obtained in three independent assays. Error bars represent S.D. between replicates. Data points with no error bars indicate that the variation among values was 0 or close to 0.

The concentration-response curves of the leaf and root extracts from five chicory cultivars in the anti-protozoal assays with extracellular *T. cruzi* trypomastigotes are presented in Fig. 2. All tested extracts induced a concentration-dependent activity against *T. cruzi* trypomastigotes, but with differences between cultivars and leaf and root extracts. Root extracts induced a higher anti-protozoal activity than the leaf extracts from the same cultivar, with significantly lower EC_50_ (P < 0.001; Table 2), except for the Maestoso-Leaf extract that exerted a more potent trypanocidal effect in comparison with the Maestoso-Root extract (P < 0.0001; Table 2). The Larigot-Root and the Spadona-Leaf extracts induced the highest and lowest trypanocidal activity, respectively.

**Table 2:** Effective concentration able to induce 50% inhibition of viability of *Trypanosoma cruzi* trypomastigotes (EC_50_) or to reduce the viability in 50% of uninfected Vero cells (CC_50_) after 24 h of incubation with purified extracts from leaves and roots from five *Cichorium intybus* cultivars.

Cultivar	Plant part	*T. cruzi* trypomastigotes	R^2^	Vero cells	Selectivity Index
		EC_50_^a^ (95% CI)		CC_50_^b^ (95% CI)	(CC_50_ Vero cells/EC_50_*T. cruzi*)
Benulite	Leaf	38.18 (36.4–40.3)	0.97	191.6 (160.2–245.0)***	5.01
	Root	32.53 (30.3–34.8)**	0.95	96.3 (94.8–97.7)	2.96
Goldine	Leaf	36.18 (34.4–38.0)	0.96	217.1 (183.1–275.1)***	6.00
	Root	27.87 (26.7–29.1)***	0.98	73.24 (69.3–77.3)	2.63
Larigot	Leaf	34.3 (32.3–36.4)	0.96	180.3 (138.7–327.1)***	5.26
	Root	20.16 (18.8–21.6)***	0.94	89.47 (85.5–93.4)	4.44
Maestoso	Leaf	31 (30.1–32.0)***	0.98	382.2 (191.3–9968)***	12.33
	Root	41.3 (38.7–43.9)	0.95	102.1 (99.2–105.5)	2.47
Spadona	Leaf	41.46 (38.3–44.7)	0.97	3733 (706.3–62041)***	90.04
	Root	29.79 (28.2–31.4)***	0.93	68.28 (65.3–71.5)	2.29

Statistical difference between purified leaf and root extract within the same cultivar: ***P < 0.0001: **P = 0.0002; CI = confidence interval; R^2^ = goodness-of-fit of concentration-response curves for *T. cruzi* trypomastigotes.

a Effective concentration able to reduce the viability in 50% of the exposed *T. cruzi* trypomastigotes (EC_50_) in μg extract/mL.

b Cytotoxic concentration able to reduce the viability in 50% of the exposed Vero cells (CC_50_) in μg extract/mL.

### 3.3 Cytotoxicity of purified *C. intybus* extracts on uninfected Vero cells

The cytotoxicity of *C. intybus* on uninfected mammalian Vero cells and the selectivity index (SI) of each tested extract against *T. cruzi* are presented in Table 2. The mean cell viability of Vero cells in the negative control (0.1% DMSO) at 24 h incubation was 99.33% (95% CI = 98.4–100). The leaf extracts were significantly less cytotoxic towards Vero cells (higher CC_50_) than the root extracts from all *C. intybus* cultivars (P < 0.0001). All the root extracts at the highest concentration tested (100 μg/mL) induced a marked cytotoxic effect and reduced the viability of exposed uninfected Vero cells below 50% (mean cell viability [95% CI] = 37.03% [33.1–40.9]). In contrast, the mean viability of Vero cells exposed to *C. intybus* leaf extracts at the highest concentration (100 μg/mL for 24 h) was ≥80% (95% CI = 77.8–81.9). This lower cytotoxicity resulted in a higher SI of purified *C. intybus* leaf extracts against *T. cruzi*, in comparison to the root extracts (Table 2). The Spadona-leaf extract had the highest trypanocidal SI (90.04), followed by the Maestoso (12.33) and the Goldine (6.00) leaf extracts.

### 3.4 Anti-protozoal activity of purified *C. intybus* extracts against intracellular *T. cruzi* amastigotes

**Fig. 3:**
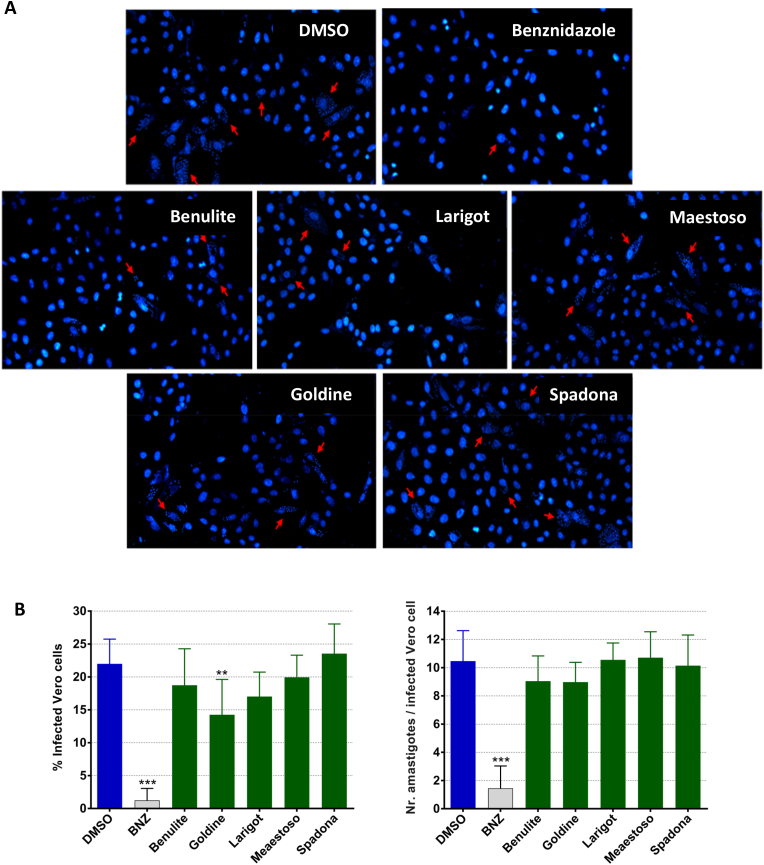
Effect of purified *Cichorium intybus* leaf extracts on intracellular *Trypanosoma cruzi* amastigotes. Vero cells were infected with *T. cruzi* trypomastigotes (Y strain) and treated with purified leaf extracts (10 μg/mL) from five *C. intybus* cultivars for 48 h. Vero cells exposed to DMSO (0.1%) or benznidazole (26 μg/mL) were run in parallel as negative and positive controls, respectively. After incubation, intracellular parasite load was assessed by DAPI staining of DNA from Vero cells and *T. cruzi* amastigotes. A) Representative fluorescence microscopy images show the effect of each treatment on intracellular amastigotes, with red arrows indicating infected Vero cells. B) Quantification of the effect of *C. intybus* leaf extracts on intracellular *T. cruzi* amastigotes. The left and right graph show the percentage of infected cells and the number of amastigotes per infected cell, respectively, analysed in three random images observed under fluorescence microscopy per replicate. Each bar represents the mean in nine replicates for each condition obtained in three independent assays. Error bars represent S.D. between replicates. ***P < 0.0001; **P < 0.01. (For interpretation of the references to colour in this figure legend, the reader is referred to the Web version of this article.)

The trypanocidal activity of the *C. intybus* leaf extracts (higher SI) was further tested on Vero cells infected with intracellular *T. cruzi* amastigotes (Fig. 3). Representative fluorescence microscopic images of Vero cells infected with *T. cruzi* amastigotes and exposed to *C. intybus* extracts at 10 μg/mL (or to negative/positive controls) are depicted in Fig. 3A and were further analysed for quantification of treatment effects. The leaf extract from *C. intybus* cv. Goldine was the only able to significantly reduce the number of infected Vero cells by 35.2%, in comparison with the negative control (P = 0.0013; Fig. 3B). Leaf extracts from *C. intybus* cv. Larigot, Benulite and Maestoso also decreased the number of infected cells by 22.6%, 14.8% and 9.3%, respectively, in comparison with infected cells exposed to 0.1% DMSO (P > 0.06), while exposure to the Spadona-leaf extract did not affect the number of infected Vero cells (Fig. 3B). None of the tested leaf extracts significantly reduced the number of amastigotes per infected cell. The extracts from *C. intybus* cv. Goldine and cv. Benulite marginally decreased the number of amastigotes per cell by 14.3% and 13.6%, respectively, in comparison with the negative control (P > 0.1; Fig. 3B). In contrast, the positive control BNZ (tested at 26 μg/mL) induced a significant reduction in the number of infected cells and the number of amastigotes per infected cell by 94.4% and 86.2%, respectively (P < 0.0001; Fig. 3B).

### 3.5 Bioactivity-based molecular networking for prediction of trypanocidal compounds in purified *C. intybus* extracts

**Fig. 4:**
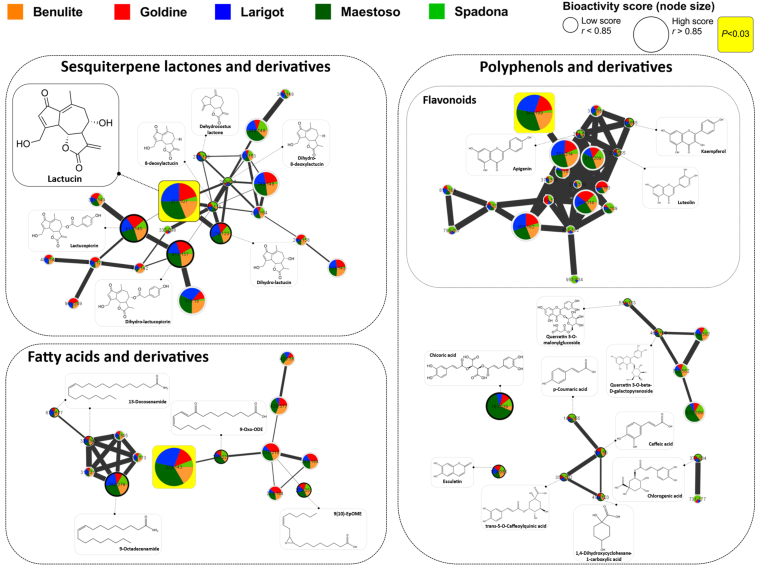
Predicted trypanocidal compounds from *Cichorium intybus* against *Trypanosoma cruzi* trypomastigotes. Bioactivity-based molecular network of sesquiterpene lactones, polyphenols and fatty acids and their derivatives in five purified *C. intybus* leaf extracts based on their activity against extracellular *T. cruzi* trypomastigotes. Each node represents one molecule detected by UHPLC-HRMS in *C. intybus*, with the molecular weight of its precursor ion inside the node. Edges (connections) between nodes represent the spectrum-to-spectrum alignment between two compounds in relation with their fragmentation pattern (i.e. the thicker the connection, the more related the compounds are). Pie charts inside nodes describe the relative concentration (based on peak area) of each molecule among the different extracts. Node sizes proportionally reflect the predicted bioactivity score of the molecule. The bioactivity score is the Pearson correlation coefficient (*r*) between the molecule's relative abundance (peak area) and the EC_50_ of each extract against *T. cruzi* trypomastigotes (Table 2). The nodes with yellow squares represent molecules with statistically significant high bioactivity scores (*r* > 0.85 with P < 0.03). Identified compounds (library hits) are presented as nodes with their chemical structures. Unidentified derivatives (no-hit in the libraries but grouped within a specific class of compounds) are only presented as nodes. Please see Supplementary Fig. 12 for the full bioactivity-based molecular network including identified compounds, unidentified derivatives and not annotated molecules. (For interpretation of the references to colour in this figure legend, the reader is referred to the Web version of this article.)

**Fig. 5:**
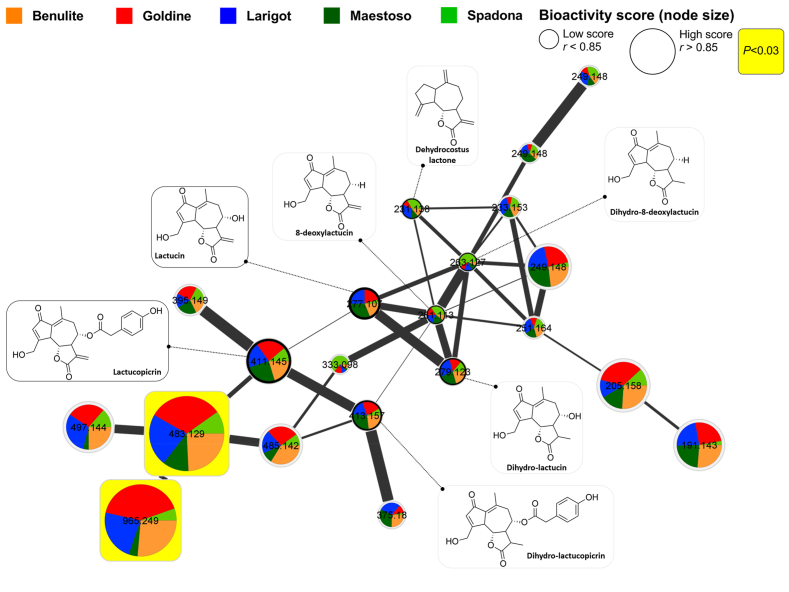
Predicted trypanocidal compounds from *Cichorium intybus* against *Trypanosoma cruzi* amastigotes. Bioactivity-based molecular network of sesquiterpene lactones (SL) and SL-derivatives in five purified *C. intybus* leaf extracts based on their activity against intracellular *T. cruzi* amastigotes. Each node represents one molecule detected by UHPLC-HRMS in *C. intybus*, with the molecular weight of its precursor ion inside the node. Edges (connections) between nodes represent the spectrum-to-spectrum alignment between two compounds in relation with their fragmentation pattern (i.e. the thicker the connection, the more related the compounds are). Pie charts inside nodes describe the relative concentration (based on peak area) of each molecule among the different extracts. Node sizes proportionally reflect the predicted bioactivity score of the molecule. The bioactivity score is the Pearson correlation coefficient (*r*) between the molecule's relative abundance (peak area) and the percentage reduction of infected cells with *T. cruzi* amastigotes by each purified extract. The nodes with yellow squares represent molecules with statistically significant high bioactivity scores (*r* > 0.85 with P < 0.03). Identified SL (library hits) are presented as nodes with their chemical structures. Unidentified SL-derivatives are only presented as nodes. Please see Supplementary Fig. 13 for the full bioactivity-based molecular network including identified compounds, unidentified derivatives and not annotated molecules. (For interpretation of the references to colour in this figure legend, the reader is referred to the Web version of this article.)

To further explore the relationship between the compounds present in the five *C. intybus* leaf extracts (higher selectivity towards *T. cruzi*), bioactivity-based molecular networking analyses were performed to facilitate the prediction and identification of trypanocidal molecules towards extracellular trypomastigotes (Fig. 4) and intracellular amastigotes (Fig. 5). A bioactivity-based molecular network represents the relationship between structurally-related metabolites in the analysed extracts, and predicts the bioactivity of each molecule in those extracts. In Figs. 4 and 5, each node represents the spectra of one molecule, and pie charts inside nodes illustrate the relative concentration of each molecule among all *C. intybus* leaf extracts. The edges between nodes correspond to the spectrum-to-spectrum alignment between two compounds in relation to their fragmentation pattern. All identified compounds (library hits) and their derivatives were clustered within one of three classes of chemical compounds in the molecular network: SL, polyphenols or fatty acids.

First, we performed a molecular networking analysis by computing the bioactivity scores of each compound based on the bioactivities (EC_50_) of the purified leaf extracts against extracellular *T. cruzi* trypomastigotes (Fig. 4). The five *C. intybus* leaf extracts induced a concentration-dependent effect against *T. cruzi* trypomastigotes, but with different EC_50_ between extracts (Table 2). These EC_50_ values (normally distributed; Shapiro-Wilk normality test P > 0.9) were used for bioactivity score prediction by calculating the Pearson coefficient of correlation between the EC_50_ of the leaf extracts with the normalized peak intensity of each compound detected in the extracts (total 196 metabolites). In Fig. 4, the node sizes proportionally reflect the bioactivity score (Pearson correlation coefficient *r*) of each molecule, with yellow squares surrounding nodes indicating molecules with a significantly high bioactivity score, and thus a predicted high trypanocidal activity towards *T. cruzi* trypomastigotes (*r* > 0.85 with P < 0.03). The bioactivity-based molecular networking analysis resulted in 11 compounds with a significantly high bioactivity score, meaning that the increased concentration of these metabolites had a strong and significant positive correlation with higher anti-protozoal activity against *T. cruzi* trypomastigotes. From the molecules with high bioactivity score, the only identified compound (library hit) was the SL lactucin (molecular weight of precursor ion [MW] = 277.107; *r* = 0.94; P = 0.017). Two other unidentified compounds (no library hit) clustered within the polyphenols (flavonoid compound with MW = 349.199; *r* = 0.94; P = 0.018) and fatty acids (molecule related with 9-Oxo-ODE with MW = 229.143; *r* = 0.96; P = 0.008) were also predicted to have significantly high bioactivity scores (Fig. 4). Moreover, eight not annotated compounds (not clustered in any of the mentioned chemical classes) also had significantly high bioactivity scores (Supplementary Fig. 12). Next, we performed a second molecular networking analysis by calculating the bioactivy scores of each compound based on the percentage reduction of infected Vero cells with *T. cruzi* amastigotes induced by the *C. intybus* leaf extracts (Fig. 5). This analysis resulted in seven compounds with a significantly high bioactivity score (*r* > 0.85; P < 0.03), including two unidentified SL-derivatives (MW = 483.129; *r* = 0.93; P = 0.021) and MW = 965.249; *r* = 0.91; P = 0.029; Fig. 5) and five not annotated compounds (Supplementary Fig. 13).

## 4 Discussion

In the present study, we have described anti-protozoal effects of *C. intybus* against *T. cruzi* trypomastigotes, with a higher trypanocidal selectivity and lower cytotoxicity on mammalian cells of purified leaf extracts. In addition, the leaf extract from *C. intybus* cv. Goldine significantly reduced the number of cells infected with *T. cruzi* amastigotes. Untargeted metabolomic and bioactivity-based molecular networking analyses revealed 11 compounds strongly linked with the anti-parasitic activity of *C. intybus* against trypomastigotes, including the SL lactucin, and flavonoid and fatty acid derivatives. Furthermore, seven distinct *C. intybus* molecules (including two SL-derivatives) were predicted to be involved in reducing the number of Vero cells infected with *T. cruzi* amastigotes.

All extracts from the five *C. intybus* cultivars induced a concentration-dependent activity against extracellular *T. cruzi* trypomastigotes, but with different potencies between leaf and root extracts and among cultivars. Root extracts were more potent than leaf extracts of the same cultivar against trypomastigotes, with one exception: the leaf extract of *C. intybus* cv. Maestoso. A higher anti-parasitic activity of *C. intybus* Maestoso-leaf vs. root extracts was also recently reported against the parasitic nematode *Ascaris suum* (Valente et al., 2021). In the present study, the enhanced anti-protozoal activity of the Maestoso-leaf extract, in comparison with the Maestoso-root extract, was likely related with their different metabolite profiles. In this regard, the Maestoso-leaf extract had the highest concentration of chicoric acid (MW = 163.039) among leaf and root extracts from all tested *C. intybus* cultivars, as well as the largest concentrations of an unidentified sesquiterpene lactone-derivative (MW = 205.158) and two fatty acid-derivatives (MW = 229.143, which had a significantly high bioactivity score against trypomastigotes, and MW = 425.377). These compounds may be related with the marked anti-*T. cruzi* of the Maestoso-leaf extract, and further studies should confirm their role in the apparent broad anti-parasitic effects elicited by leaves from this *C. intybus* cultivar.

In addition to their potent anti-trypomastigote activity, the *C. intybus* root extracts also induced a higher toxicity on mammalian cells, in comparison with the leaf extracts, irrespective of cultivar. The lower cytotoxicity of *C. intybus* leaf extracts towards uninfected cells could be explained by i) the presence of cytotoxic molecules in *C. intybus* roots that are absent in the leaves and/or ii) the synthesis of protective compounds in *C. intybus* leaves that reduced the cytotoxicity of other metabolites in the tested extracts. Additional bioactivity-based molecular networking analyses of *C. intybus* root and leaf extracts based on their cytotoxicity (CC_50_) on uninfected Vero cells (data not shown) predicted ten not annotated molecules only in the root extracts with significantly high bioactivy scores (i.e. higher cytotoxicity) towards mammalian cells (MWs = 763.552; 691.495; 647.469; 749.538; 705.512; 719.525; 661.485; 603.443; 837.590; 389.170). In comparison, merely one not annotated compound present in the leaf extracts was predicted to have a high bioactivity score against uninfected Vero cells (MW = 207.138). Thus, the presence of several of not annotated molecules with a predicted high cytotoxicity only in *C. intybus* roots may have been related with their increased cytotoxicity in our *in vitro* model. Furthermore, the leaf extracts from all *C. intybus* cultivars had markedly higher concentrations of chlorogenic acid, caffeic acid and esculetin, compared with root extracts. These polyphenols have been reported to elicit antioxidant and protective mechanisms in different biological systems exposed to toxic agents (Sato et al., 2011; Filipský et al., 2015; Rashidi et al., 2022), and their enhanced concentration in *C. intybus* leaves may have contributed to their low toxicity by protecting the exposed cells from cytotoxic compounds.

Based on the low cytotoxicity of *C. intybus* leaves on mammalian cells, and their resulting higher anti-*T. cruzi* selectivity in comparison with roots, we tested all purified leaf extracts for their activity against intracellular *T. cruzi* amastigotes. Here, the leaf extract from *C. intybus* cv. Goldine was the only to significantly reduce the number of mammalian cells infected with *T. cruzi* amastigotes. However, none of the tested leaf extracts decreased the number of amastigotes per infected cell, which were in contrast significantly reduced by the positive control BNZ. Considering the marked trypanocidal activity and high SI of *C. intybus* leaf extracts against extracellular trypomastigotes, the lower activity against intracellular parasites may be explained by two causes. First, poor pharmacokinetics of *C. intybus* metabolites in the infected Vero cells linked to host cell factors (such as interferences in the uptake of the molecules by the plasma membrane) may have limited the cytoplasmic concentrations of the compounds that reached the intracellular parasites (Alcântara et al., 2018). Secondly, only one concentration of *C. intybus* leaf extracts was tested on infected Vero cells (10 μg extract/mL), and therefore, the poor activity on intracellular *T. cruzi* amastigotes may have been a consequence of the (low) concentration evaluated. Therefore, further studies could confirm the intracellular trypanocidal activity of *C. intybus* compounds, and study their uptake by host cells, at increased concentrations.

Bioactivity-based molecular networking analyses to infer potential trypanocidal molecules in *C. intybus* predicted 11 metabolites with a significantly high activity against *T. cruzi* trypomastigotes, and seven compounds with a significantly high activity in reducing the number of Vero cells infected with *T. cruzi* amastigotes. Interestingly, none of the seven compounds with a high bioactivity towards intracellular amastigotes were predicted to have a significant activity against extracellular trypomastigotes, suggesting that distinct *C. intybus* metabolites may act on different life stages of *T. cruzi*. However, considering that in our study none of the purified *C. intybus* extracts reduced the number of *T. cruzi* amastigotes per infected cell, these computational predictions should be considered with caution. The SL lactucin was the only identified compound with a significantly high bioactivity score activity against trypomastigotes, whereas two unidentified SL-derivatives were predicted to have a significant high activity towards amastigotes. Previously, Bischoff et al. (2004) reported that pure lactucin and lactucopicrin isolated from *C. intybus* exerted a concentration-dependent *in vitro* growth inhibition of the malarial parasite *P. falciparum*, with complete parasite growth inhibition of lactucin and lactucopicrin at 10 and 50 μg/mL, respectively (no EC_50_ reported). More recently, Woolsey et al. (2019) described the *in vitro* activity of *C. intybus* leaf and roots extracts against *C. parvum*, with leaves inducing a significantly stronger parasite growth inhibition (lower EC_50_) than roots. In that study, lactucin and dihydro-lactucin were present in an increased proportion in the more potent leaf extract compared to the root extract, although the roots had a higher concentration of individual and total SL; therefore, the SL alone were likely not the sole responsible for the higher anti-*C. parvum* activity of *C. intybus* leaves (Woolsey et al., 2019). Similarly, the observed anti-*T. cruzi* activity of the purified *C. intybus* leaf extracts in our study is probably explained by the action of several bioactive compounds. Besides SL, molecular networking analyses predicted significantly high bioactivity scores for flavonoid and fatty acid derivatives. Natural flavonoids have been extensively described to induce anti-protozoal activity against several trypanosomatid parasites (Cockram and Smith, 2018; Izumi et al., 2011), whereas fatty acids were recently suggested to be involved in the anti-parasitic activity of seaweeds (Bonde et al., 2021). Furthermore, in the present study the various not annotated compounds with high bioactivity scores likely contributed to the activity of the tested purified extracts, either as single compounds or in combination with other metabolites.

Valente et al. (2021) recently elucidated the anthelmintic molecules of *C. intybus* by molecular networking and bioguided-fractionation, confirming the SL 8-deoxylactucin as the main anti-nematodal metabolite (as single compound and in synergistic combination with other SL). In the present work, we tested similar purified *C. intybus* extracts as in Valente et al. (2021), and our analyses suggested that the compounds responsible for the anti-protozoal activity of *C. intybus* are different than those exerting anthelmintic effects, including various identified and not annotated molecules with high bioactivity scores towards *T. cruzi*. Therefore, further studies are needed to confirm the anti-protozoal metabolite(s) in *C. intybus*, for example by bioguided-fractionation of leaf material, followed by isolation and testing of purified compounds as single molecules and in combination. Additional experiments could also directly test the most promising trypanocidal compounds suggested by our molecular networking analyses, such as the SL lactucin (high bioactivity score against *T. cruzi* trypomastigotes), the two unidentified SL-derivatives with a high bioactivy in reducing the number of cells infected with *T. cruzi* amastigotes, as well as chicoric acid (possibly related with the higher trypanocidal activity of the Maestoso leaf-extract). Our present investigation thus provide a foundation for this further research that could lead to the identification of novel anti-protozoal compounds with therapeutic potential against Chagas disease and other trypanosomatid parasites, and which may also be used as starting molecules for further chemical optimization. In addition, preliminary studies by our group showed a synergistic trypanocidal activity of purified *C. intybus* leaf extracts and BNZ (Peña-Espinoza et al., unpublished observations), and further research could also explore the potential combination of *C. intybus* compounds and established anti-chagasic drugs against *T. cruzi*.

In conclusion, we have described concentration-dependent trypanocidal effects of purified *C. intybus* extracts against extracellular *T. cruzi* trypomastigotes, with *C. intybus* leaf extracts demonstrating higher anti-parasitic selectivity and lower cytotoxicity on mammalian cells than root extracts. The leaf extract of *C. intybus* cv. Goldine was also able to significantly reduce the number of mammalian cells infected with *T. cruzi* amastigotes. Metabolomic and bioactivity-based molecular networking revealed distinct compounds in *C. intybus* leaves strongly linked with activity against *T. cruzi* trypomastigotes and amastigotes, including the SL lactucin and SL-derivatives. Further studies elucidating the precise trypanocidal compound(s) and their mode(s) of anti-parasitic action will improve our knowledge on the use of *C. intybus* as a source of novel anti-trypanosomatid agents with associated health benefits and biomedical potential.

## Declaration of competing interest

The authors declare the following financial interests/personal relationships which may be considered as potential competing interests:

Matthew de Roode reports a relationship with Sensus B/V that includes: employment.
