# Supplementary material for: Anti-protozoal activity and metabolomic analyses of Cichorium intybus L. against Trypanosoma cruzi
Source: Int J Parasitol Drugs Drug Resist. 2022 Aug 13;20:43–53. doi: 10.1016/j.ijpddr.2022.08.002 (PMC9440258; doi:10.1016/j.ijpddr.2022.08.002)
Supplement: Multimedia component 1 [file mmc1.pdf]

Supplementary Table 1. List of compounds (identified and not annotated) detected in the tested *Cichorium intybus* extracts. Leaf and root extracts from *C. intybus* (cv. Benulite, cv. Goldine, cv. Larigot, cv. Maestoso and cv. Spadona) were analysed by untargeted metabolomics using UHPLC-HRMS. Metabolites were identified based on the Global Natural Product Social (GNPS) Molecular Networking libraries. Compounds are presented with their retention time in min, precursor ion mass (m/z), precursor ion intensity and compound name (for identified compounds - library hits). Not annotated molecules (no hit in GNPS libraries) are marked as "N/A".

Retention time (Consensus - min)	Precursor ion mass (m/z)	Precursor ion intensity	Compound Identification (hit on GNPS spectral libraries)
1.83	111.0153	36230.7773	Not annotated (N/A)
3.28	355.1026	84223.59659	trans-5-O-Caffeoylquinic acid
3.52	163.0396	413377.9232	Spectral Match to Chicoric acid from NIST14
3.52	377.0847	51797.0523	Spectral Match to Chlorogenic acid from NIST14
3.55	316.2121	152925.0171	N/A
3.67	731.1778	17881.66215	N/A
3.72	203.0318	369160.6097	N/A
3.73	181.0501	976158.3519	caffeic acid-emf
3.91	179.0355	324051.8935	ReSpect:PT112500 6,7-Dihydroxycoumarin|Esculetin|Cichorigenin|Aesculetin|6,7-dihydroxy-2-benzopyrone|Esculetol|Cichoriin aglycon|6,7-Dihydroxy-2H-1-benzopyran-2-one|Esculin aglycon|6,7-dihydroxychromen-2-one
4.12	576.2119	35281.83218	N/A
4.12	279.1231	646142.8568	11-B,13-Dihydro-lactucin
4.33	572.181	43103.60592	N/A
4.33	277.1079	759908.7688	Lactucin
4.53	465.1035	73886.36773	hyperoside
4.54	165.0559	64376.11253	Spectral Match to p-Coumaric acid from NIST14
4.6	551.1033	39558.6685	Quercetin 3-O-malonylglucoside
4.62	207.1383	136912.8545	N/A
4.75	555.2927	116987.5142	Spectral Match to Tributyl phosphate from NIST14
4.76	249.1482	155019.4836	N/A
4.91	685.3605	45873.63891	N/A
5.19	693.2773	28180.8139	N/A
5.21	499.1236	65357.81932	(3R,5R)-3,5-bis[[(E)-3-(3,4-dihydroxyphenyl)prop-2-enoyl]oxy]-1,4-dihydroxycyclohexane-1-carboxylic acid
5.45	333.098	145174.5131	N/A

Retention time (Consensus - min)	Precursor ion mass (m/z)	Precursor ion intensity	Compound Identification (hit on GNPS spectral libraries)
5.59	431.1675	89888.12945	N/A
6.16	389.17	42719.24683	N/A
6.2	799.3162	30985.2821	N/A
6.28	485.1422	63617.37773	N/A
6.29	261.1131	799283.3197	8-Deoxy-lactucin
6.31	965.2498	73014.16774	N/A
6.31	483.129	375837.9426	N/A
6.35	287.0557	597457.6774	Luteolin
6.37	263.1278	546687.7919	Dihydro-8-deoxy-lactucin
6.47	209.1538	128162.9066	N/A
6.57	191.143	63568.56136	N/A
6.81	287.0557	347923.4108	Massbank:PB000166 Kaempferol|3,5,7-trihydroxy-2-(4-hydroxyphenyl)chromen-4-one
7.09	271.0606	245293.6711	Massbank:PR100224 Apigenin|Apig|4',5,7-trihydroxyflavone|Apigenol|Chamomile|5,7-Dihydroxy-2-(4-hydroxyphenyl)-4-benzopyrone|Naringenin Chalcone
7.25	413.1573	229887.2293	Dihydro-lactucopicrin
7.31	411.145	1082245.534	Lactucopicrin
7.31	317.0666	120360.582	N/A
7.47	205.1584	57741.98532	N/A
7.74	349.199	216231.2731	N/A
7.74	327.2167	132466.6914	N/A
7.75	309.2062	110079.1463	N/A
7.75	251.1643	120734.3025	N/A
7.77	675.4059	71655.87713	N/A
7.81	233.1535	167735.1455	N/A
7.85	249.1484	107211.5563	N/A
8.04	231.1382	161672.6835	MoNA:3475533 Dehydrocostus lactone
8.17	1023.6294	45670.25957	N/A
8.17	211.1332	275839.654	N/A
8.2	351.2145	338927.8558	N/A
8.2	679.438	225773.4435	N/A
8.2	229.1437	126282.6455	N/A
8.2	204.6069	97486.3284	N/A
8.24	249.1849	70437.47254	N/A

Retention time (Consensus - min)	Precursor ion mass (m/z)	Precursor ion intensity	Compound Identification (hit on GNPS spectral libraries)
8.68	249.1486	317079.5852	N/A
9.17	497.1442	41713.3435	N/A
9.17	395.1495	73534.04882	N/A
9.21	299.1974	48699.62711	N/A
9.31	409.1627	462900.4441	N/A
9.39	795.3355	197998.9347	N/A
9.39	387.1809	338516.2289	N/A
9.39	811.3095	88165.99974	N/A
9.39	790.3799	45958.74254	N/A
9.39	404.2076	129627.6395	N/A
9.45	273.1849	143104.4585	N/A
9.46	387.1851	269566.3308	N/A
9.48	375.1807	66411.08044	N/A
9.5	295.1673	148372.7305	N/A
9.61	343.1533	60382.69761	N/A
9.62	409.1705	237413.564	N/A
9.64	795.3572	27689.26208	N/A
9.7	277.2166	181632.9547	N/A
9.87	951.3348	16553.34597	N/A
9.87	487.1622	114781.911	N/A
9.92	297.1832	93384.97859	N/A
10.4	357.1696	34691.06612	N/A
11.39	295.2272	216524.1642	Spectral Match to 9-Oxo-10E,12Z-octadecadienoic acid from NIST14
11.4	317.2091	132341.9781	N/A
11.73	440.41	251790.1301	N/A
11.91	540.4262	169498.8376	N/A
12.12	376.3185	396340.0535	N/A
12.48	468.4413	228734.977	N/A
12.52	376.3188	300993.9954	N/A
12.59	861.5369	104549.6789	N/A
12.6	725.6167	103358.8197	N/A
12.63	568.4572	177444.1039	N/A
12.68	279.2319	127708.7483	Spectral Match to 9(10)-EpOME from NIST14

Retention time (Consensus - min)	Precursor ion mass (m/z)	Precursor ion intensity	Compound Identification (hit on GNPS spectral libraries)
12.88	661.3031	48167.49973	N/A
12.89	425.3776	170635.2991	N/A
12.96	282.2794	121428.0356	Spectral Match to 9-Octadecenamide, (Z)- from NIST14
13.13	691.4958	39705.58589	N/A
13.17	647.4696	42795.29857	N/A
13.17	443.3882	35557.82657	betulin
13.21	603.4434	49359.02005	N/A
13.22	845.4136	11102.73026	N/A
13.25	975.5665	67884.59595	N/A
13.26	559.4174	60833.18355	N/A
13.29	837.5908	30660.16897	N/A
13.31	515.3913	71636.45478	N/A
13.37	749.538	51070.78532	N/A
13.41	705.5124	49791.85631	N/A
13.44	625.2664	22684.77558	N/A
13.45	609.2711	28920.15565	N/A
13.46	661.4857	52065.57891	N/A
13.47	609.2711	32405.08185	N/A
13.48	511.2095	126433.5692	N/A
13.48	489.2275	35566.05527	N/A
13.48	547.2807	95656.55546	N/A
13.5	691.3139	166551.8195	N/A
13.52	617.4598	55453.41616	N/A
13.53	383.3144	68585.8852	N/A
13.58	573.434	60670.33998	N/A
13.65	529.4073	50622.66096	N/A
13.65	423.3607	49992.47044	N/A
13.65	763.5525	33796.62562	N/A
13.7	719.5256	33808.27597	N/A
13.73	310.3114	655864.2922	N/A
13.79	1295.587	98576.78019	N/A

Retention time (Consensus - min)	Precursor ion mass (m/z)	Precursor ion intensity	Compound Identification (hit on GNPS spectral libraries)
13.81	1273.604	127152.4406	N/A
13.81	1290.6303	56929.10018	N/A
13.82	654.3324	221517.6235	N/A
13.83	441.3602	51975.84918	N/A
13.91	813.5127	82507.84973	N/A
13.95	593.2761	181393.9644	N/A
13.97	659.2876	221137.0166	N/A
14.06	811.4985	48335.07752	N/A
14.11	695.359	67676.71609	N/A
14.13	695.3591	77479.05438	N/A
14.34	754.5428	106438.1266	N/A
14.34	732.5622	123951.431	N/A
14.36	989.5784	52764.58628	N/A
14.41	338.3434	2536904.334	Spectral Match to 13-Docosenamide, (Z)- from NIST14
14.41	675.6778	583977.7167	Spectral Match to 13-Docosenamide, (Z)- from NIST14
14.41	339.3459	946331.3912	N/A
14.48	954.6156	82994.96691	N/A
14.49	960.5736	172166.7134	N/A
14.49	959.5699	307728.1416	N/A
14.53	613.4823	114408.6783	N/A
14.57	842.1026	23669.36711	N/A
14.57	844.5814	21173.6475	N/A
14.71	873.6023	25142.04117	N/A
14.77	638.3387	56288.00147	N/A
14.78	613.4825	91961.1081	N/A
14.82	987.5654	61842.71623	N/A
14.82	716.5677	137697.0717	N/A
14.82	738.549	108675.1467	N/A
14.83	827.529	70754.07441	N/A
14.88	833.5892	42096.89334	N/A
14.9	621.3105	40226.28206	N/A
14.9	1263.5958	35067.16979	N/A
14.97	1572.048	83264.94492	N/A

Retention time (Consensus - min)	Precursor ion mass (m/z)	Precursor ion intensity	Compound Identification (hit on GNPS spectral libraries)
14.98	366.3731	100096.5371	N/A
14.99	643.2929	128884.6476	N/A
15.02	932.638	44005.17143	N/A
15.02	570.5091	95462.1012	N/A
15.04	792.5613	158915.0581	N/A
15.05	937.5864	118895.0874	N/A
15.11	958.6859	20757.82989	N/A
15.13	797.5181	302562.7345	N/A
15.14	798.5213	156202.742	N/A
15.33	799.5318	42915.3229	N/A
15.36	939.6016	118906.0372	N/A
15.47	683.4639	48096.91577	N/A
15.47	629.5105	29200.9782	N/A
15.49	429.3731	224608.0645	N/A
15.6	429.3727	198989.1814	N/A
15.7	629.4761	48981.81866	N/A
15.7	638.5701	35250.42313	N/A
15.82	643.5271	180913.8482	N/A
15.86	679.3639	51003.47577	N/A
15.87	777.5495	39675.26606	N/A
15.87	711.4959	44866.16484	N/A
15.89	489.4031	41259.75872	N/A
15.96	559.5174	154281.7944	N/A
16.08	1073.7337	22977.89131	N/A
16.19	611.5009	25860.36496	N/A
16.42	680.4808	88232.62683	N/A
16.43	764.5355	392183.1325	N/A
16.43	1342.9281	140607.9848	N/A
16.43	1347.8836	180793.9176	N/A
16.44	1325.9017	36344.85111	N/A
16.46	685.4361	179760.8202	N/A
16.48	708.5116	236529.5353	N/A
16.51	763.5184	235428.4044	N/A

Retention time (Consensus - min)	Precursor ion mass (m/z)	Precursor ion intensity	Compound Identification (hit on GNPS spectral libraries)
17.47	871.5753	199607.0372	Pheophytin A
17.5	871.5749	128127.7617	Pheophytin A
