# Supplementary material for: Anti-protozoal activity and metabolomic analyses of Cichorium intybus L. against Trypanosoma cruzi
Source: Int J Parasitol Drugs Drug Resist. 2022 Aug 13;20:43–53. doi: 10.1016/j.ijpddr.2022.08.002 (PMC9440258; doi:10.1016/j.ijpddr.2022.08.002)

**Supplementary Figure 1.** LC-MS/MS chromatogram of Benulite-Leaf extract


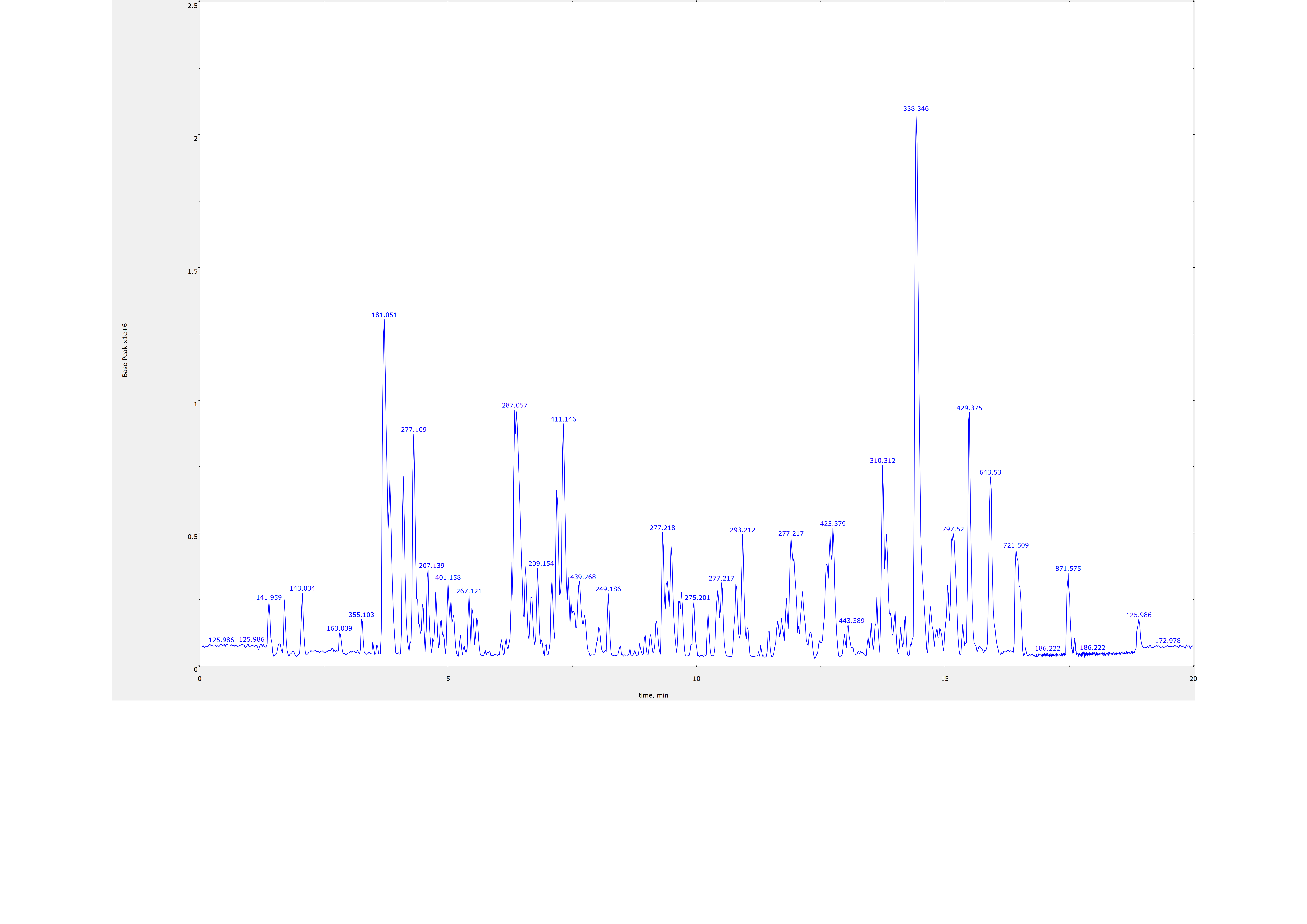


**Supplementary Figure 2.** LC-MS/MS chromatogram of Benulite-Root extract


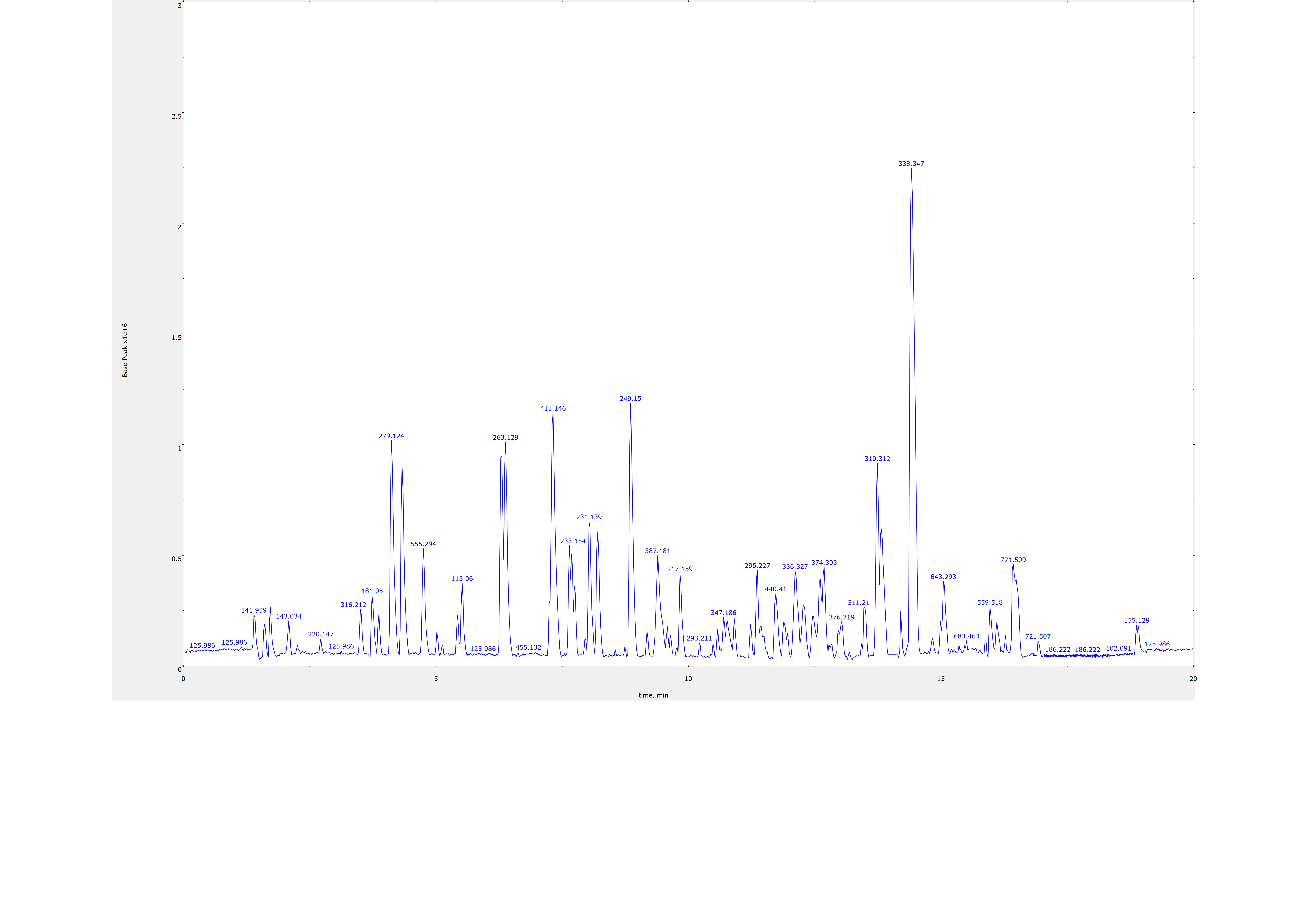


**Supplementary Figure 3.** LC-MS/MS chromatogram of Goldine-Leaf extract


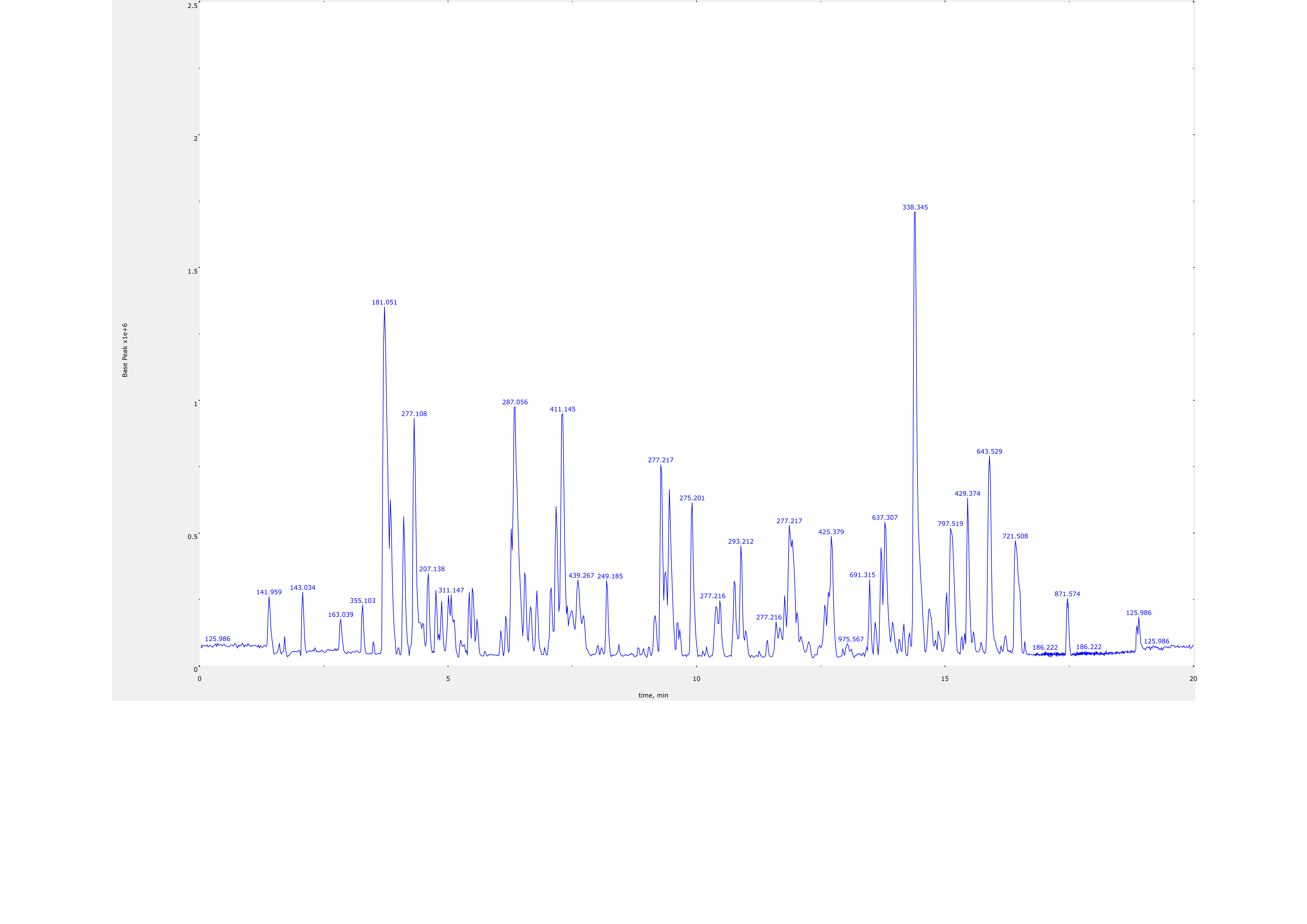


**Supplementary Figure 4.** LC-MS/MS chromatogram of Goldine-Root extract


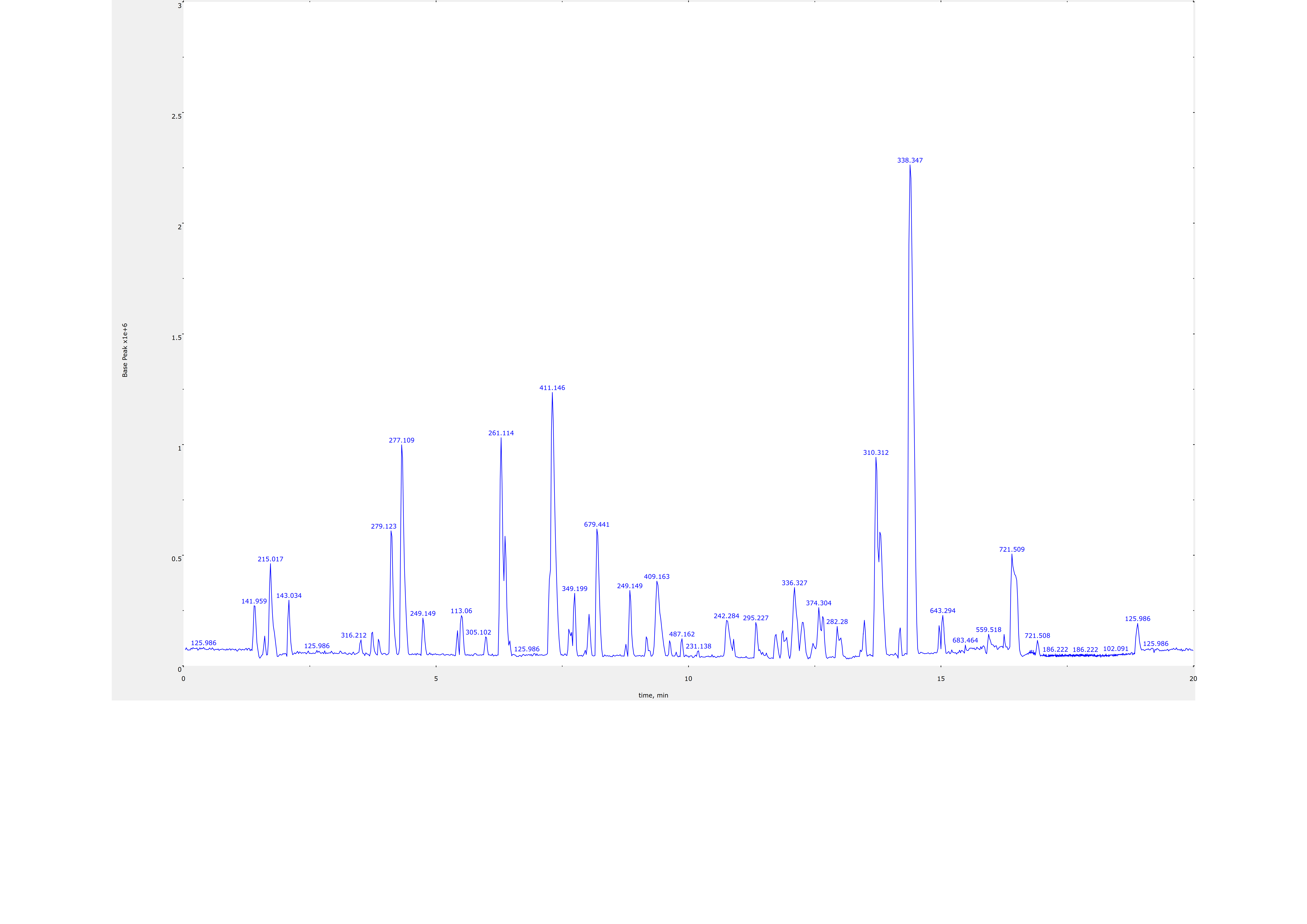


**Supplementary Figure 5**. LC-MS/MS chromatogram of Larigot-Leaf extract


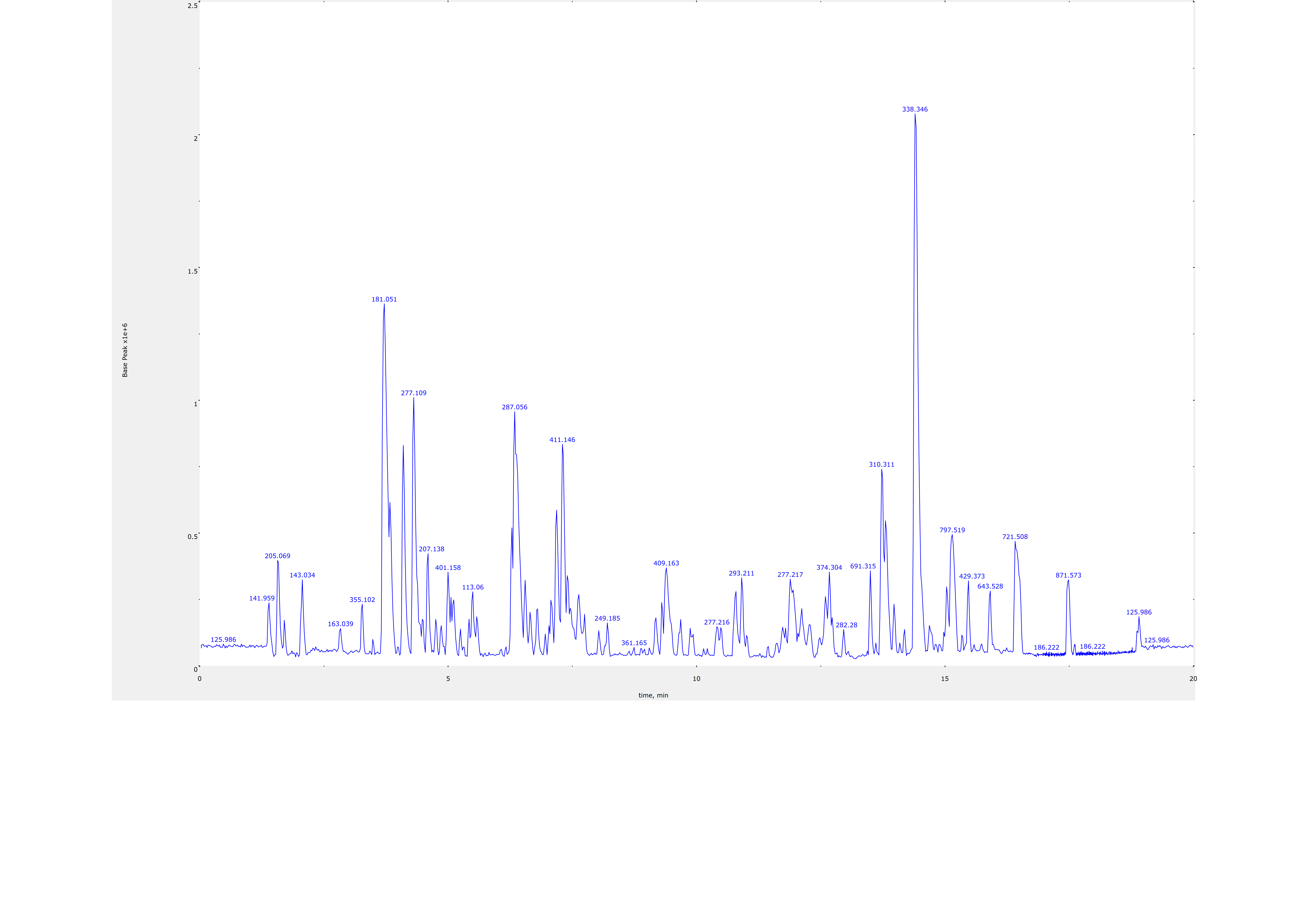


**Supplementary Figure 6**. LC-MS/MS chromatogram of Larigot-Root extract


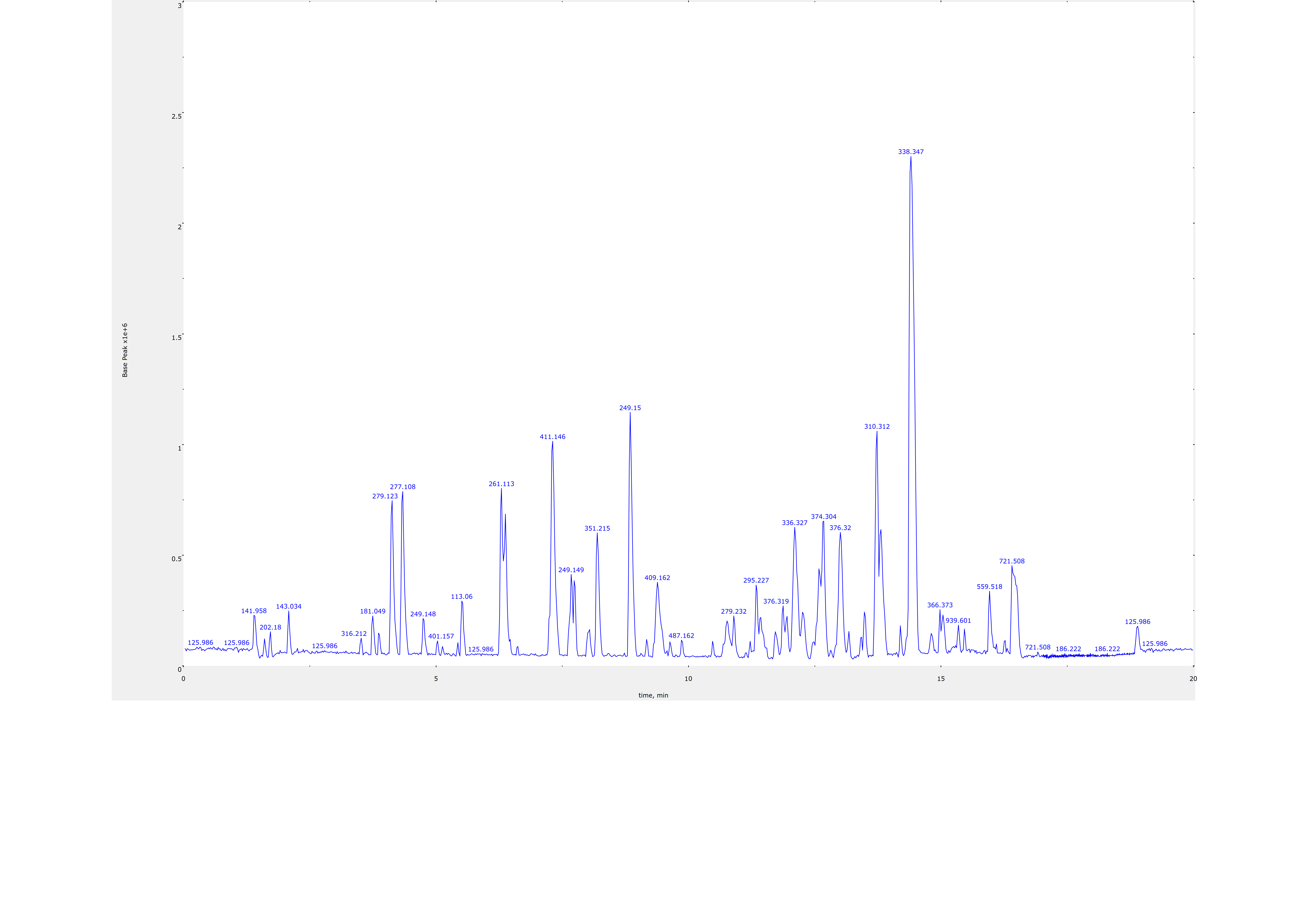


**Supplementary Figure 7.** LC-MS/MS chromatogram of Maestoso-Leaf extract


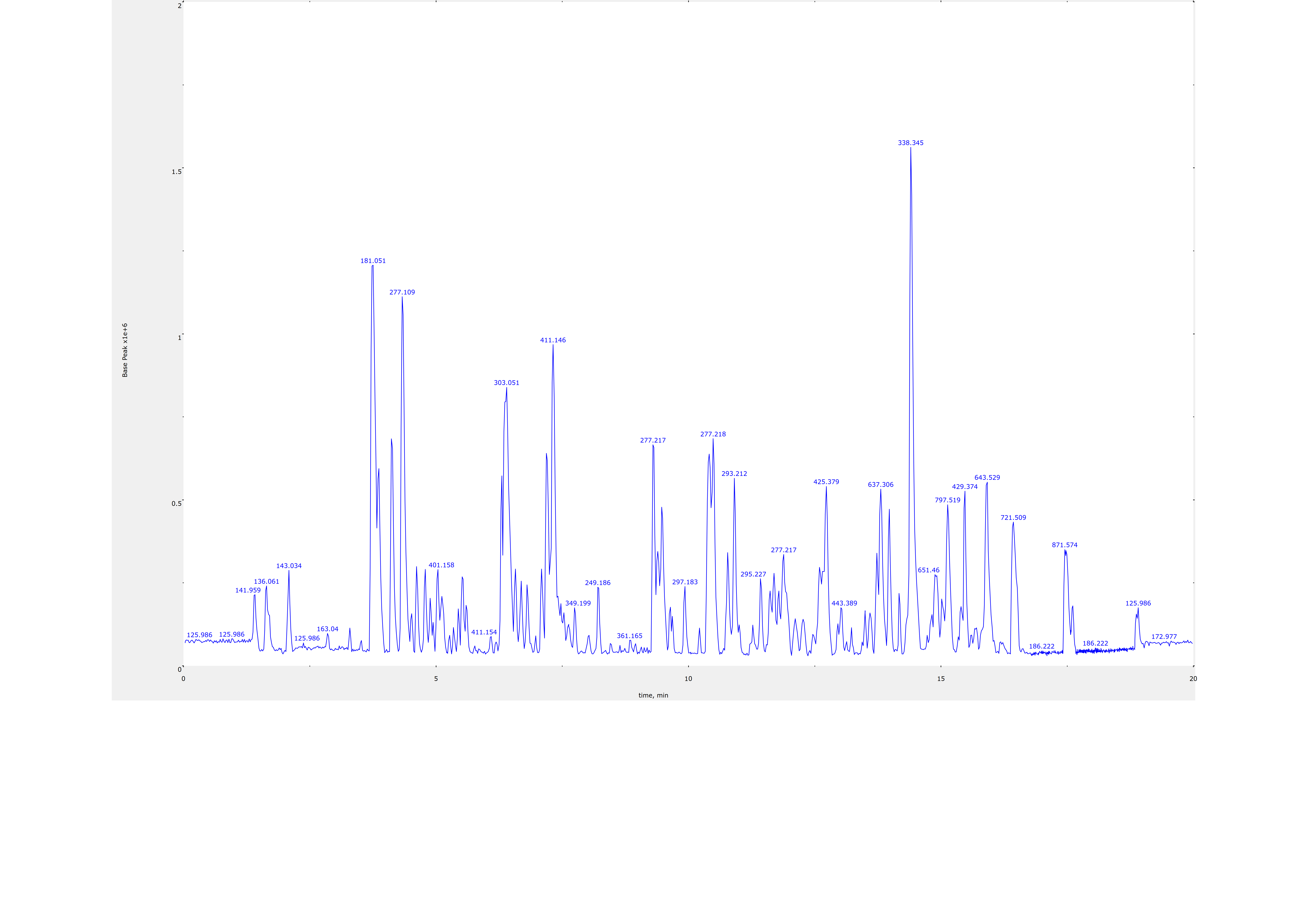


**Supplementary Figure 8.** LC-MS/MS chromatogram of Maestoso-Root extract


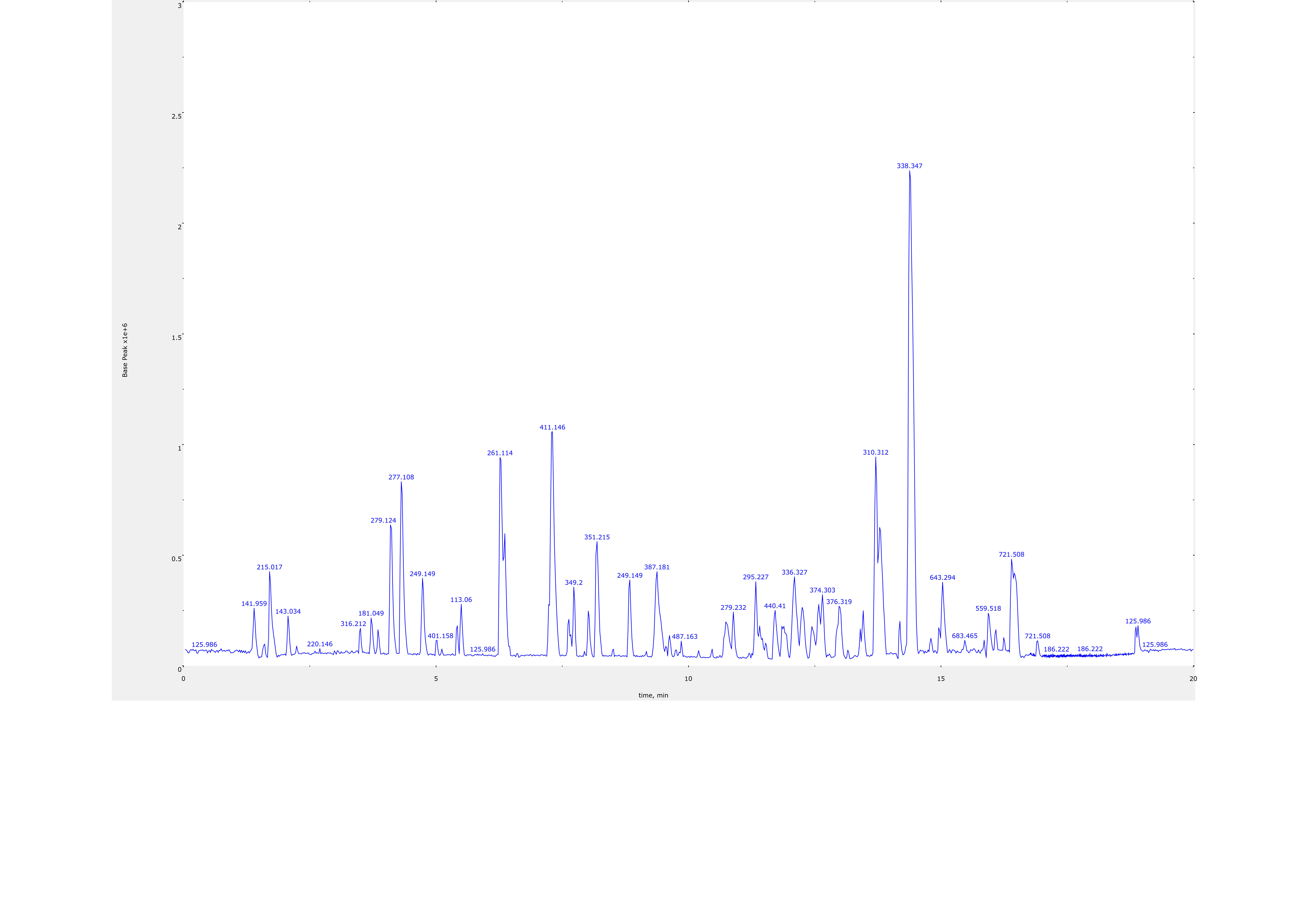


**Supplementary Figure 9.** LC-MS/MS chromatogram of Spadona-Leaf extract


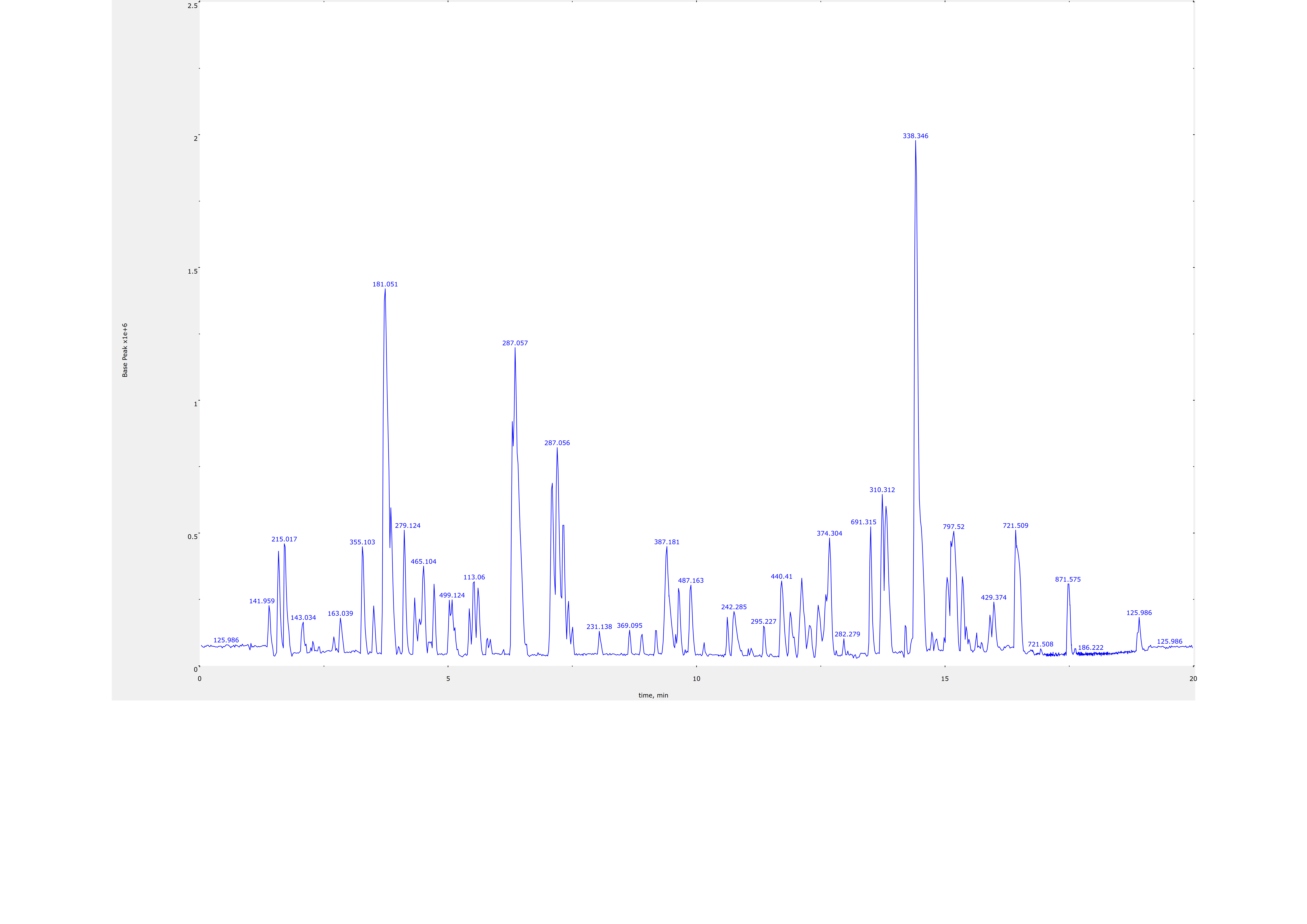


**Supplementary Figure 10.** LC-MS/MS chromatogram of Spadona-Root extract


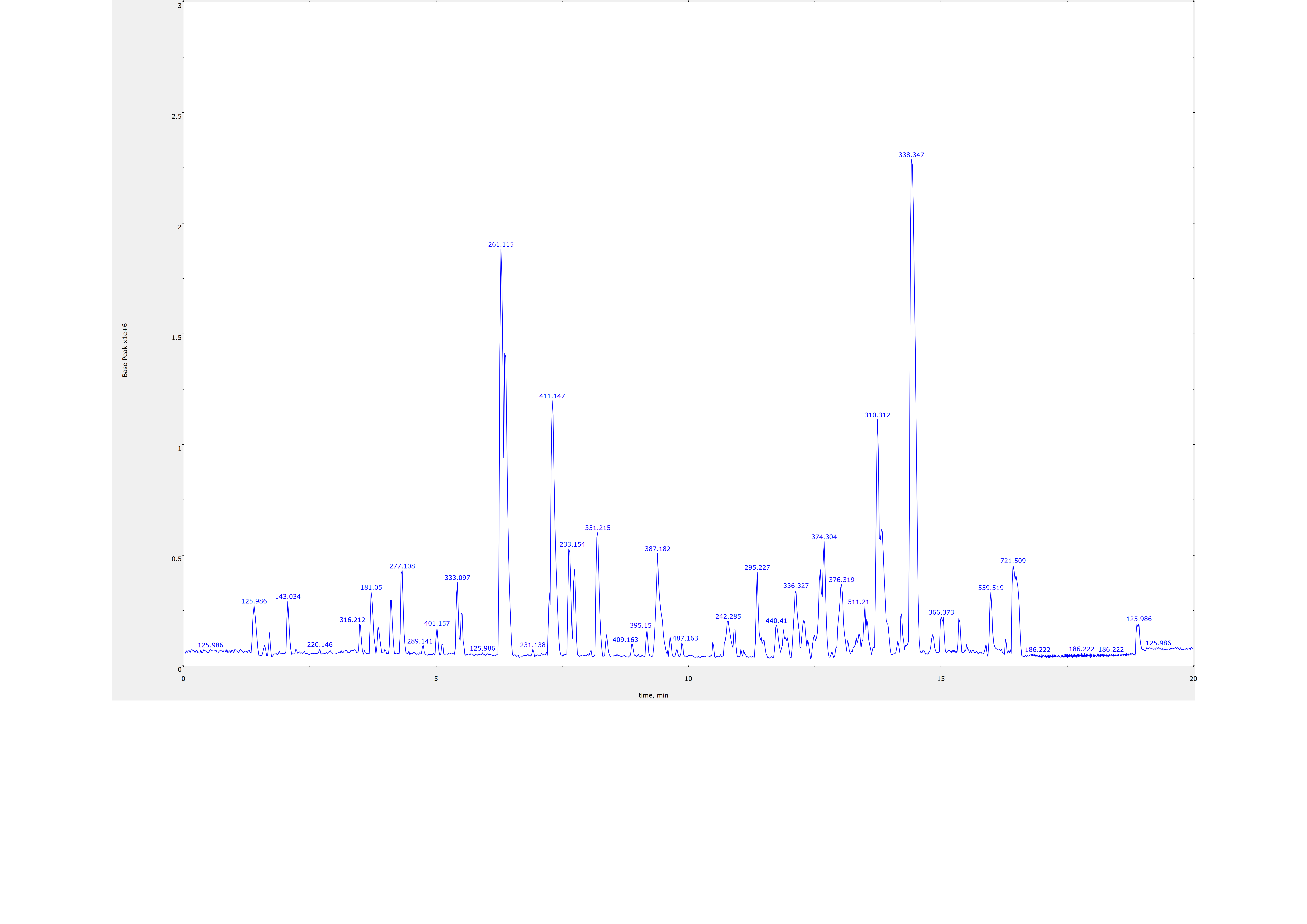


**Supplementary Figure 11.** Metabolite profile and relative quantification of not annotated molecules in purified *Cichorium intybus* extracts evaluated for trypanocidal activity. Leaf (L) and root (C) extracts from *C. intybus* (cv. Benulite, cv. Goldine, cv. Larigot, cv. Maestoso and cv. Spadona) were analysed by untargeted metabolomics using UHPLC-HRMS. These not annotated metabolites could be neither identified (no hit) in the Global Natural Product Social Molecular Networking libraries, nor grouped within a specific class of compounds (e.g. as derivative) by molecular networking. The relative quantification of the molecules is based on the peak area of the precursor ion and is illustrated in the heatmap as shades of green (with darker green representing more abundant compounds in the extracts). The unidentified (not annotated) molecules (U) are presented with their molecular weights (in Da).


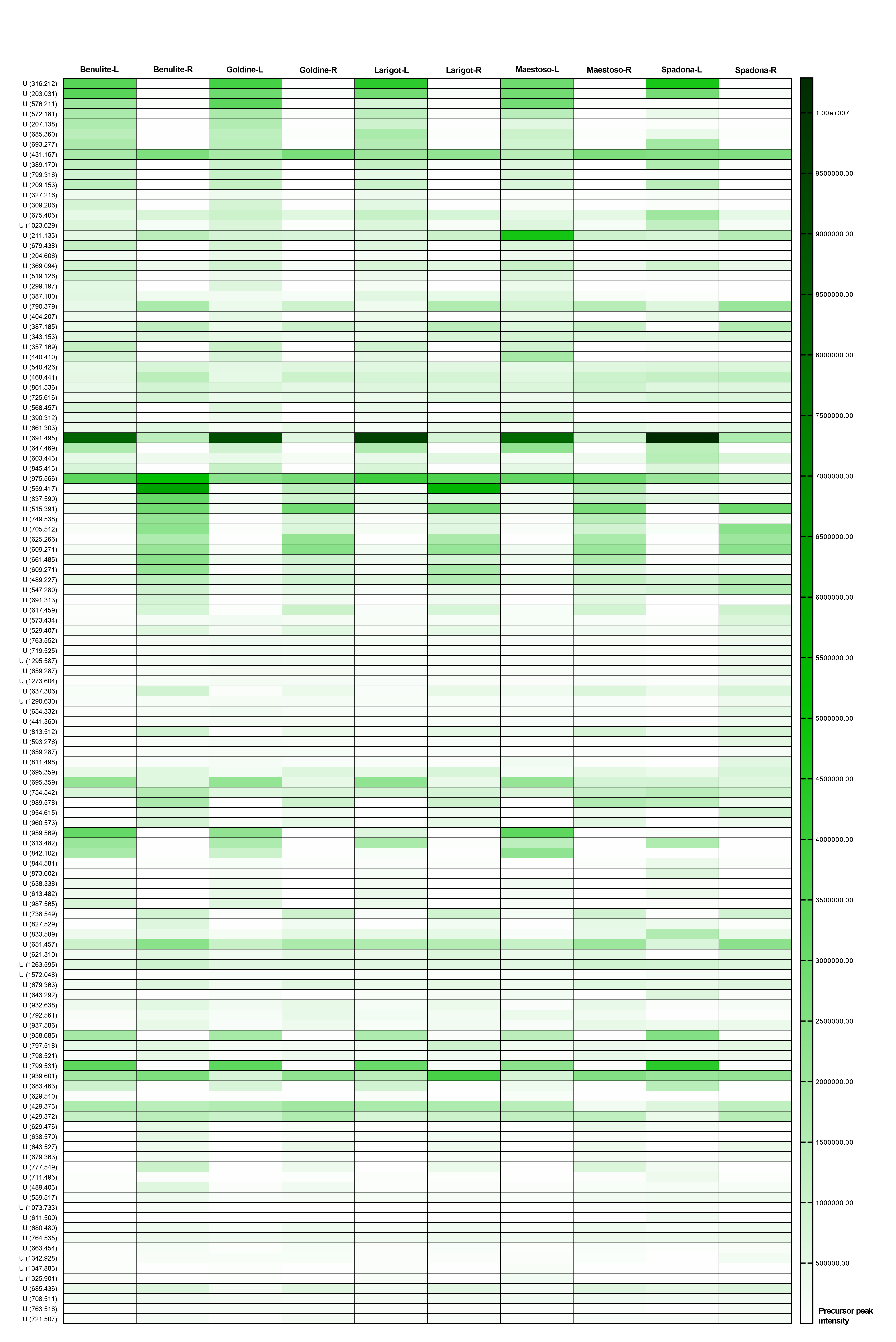


**Supplementary Figure 12.** Bioactivity-based molecular network of purified leaf extracts from *Cichorium intybus* based on their activity against *Trypanosoma cruzi* trypomastigotes. Each node represents one molecule detected by UHPLC-HRMS in *C. intybus*, with the molecular weight of its precursor ion inside the node. Edges (connections) between nodes represent the spectrum-to-spectrum alignment between two compounds in relation with their fragmentation pattern (i.e. the thicker the connection, the more related the compounds are). Pie charts inside nodes describe the relative concentration (based on peak area) of each molecule among the different extracts. Node sizes proportionally reflect the predicted bioactivity score of the molecule. The bioactivity score is the Pearson correlation coefficient (*r*) between the molecule’s relative abundance (peak area) and the EC_50_ of each extract against *T. cruzi* trypomastigotes (Table 2). The nodes with yellow squares represent molecules with statistically significant high bioactivity score (*r* > 0.85 and a significance of P < 0.03). Identified compounds (library hits) are presented as nodes with their chemical structures. Unidentified derivatives (no library hit but grouped within a specific class of compounds) and not annotated molecules are only presented as nodes.


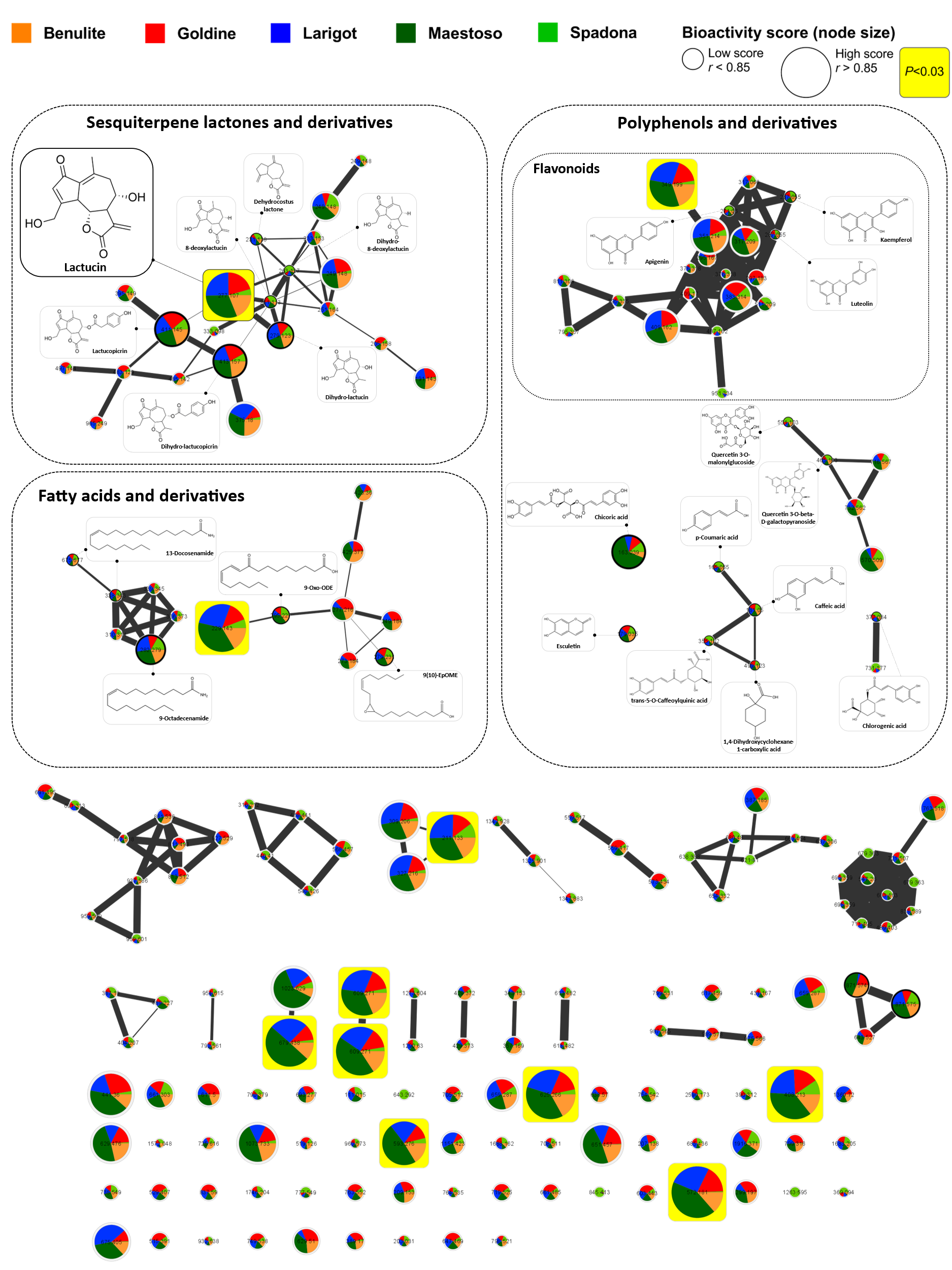


**Supplementary Figure 13.** Bioactivity-based molecular network of purified leaf extracts from *Cichorium intybus* based on their activity against *Trypanosoma cruzi* amastigotes. Each node represents one molecule detected by UHPLC-HRMS in *C. intybus*, with the molecular weight of its precursor ion inside the node. Edges (connections) between nodes represent the spectrum-to-spectrum alignment between two compounds in relation with their fragmentation pattern (i.e. the thicker the connection, the more related the compounds are). Pie charts inside nodes describe the relative concentration (based on peak area) of each molecule among the different extracts. Node sizes proportionally reflect the predicted bioactivity score of the molecule. The bioactivity score is the Pearson correlation coefficient (*r*) between the molecule’s relative abundance (peak area) and the percentage reduction of infected cells with *T. cruzi* amastigotes by each purified extract. The nodes with yellow squares represent molecules with statistically significant high bioactivity score (*r* > 0.85 and a significance of P<0.03). Identified compounds (library hits) are presented as nodes with their chemical structures. Unidentified derivatives (no library hit but grouped within a specific class of compounds) and not annotated molecules are only presented as nodes.


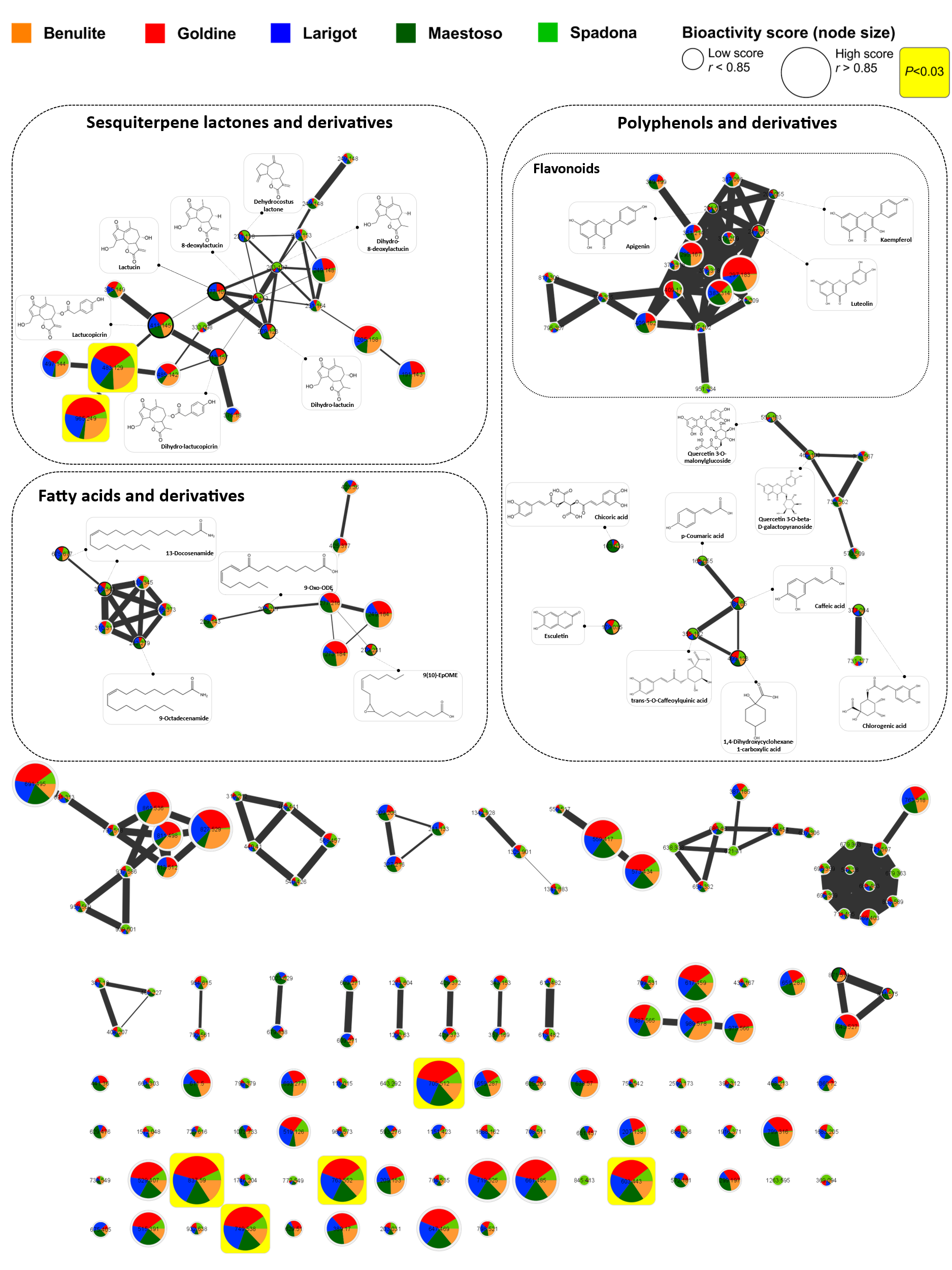

Supplement: Multimedia component 2 [file mmc2.docx]
